# Investigating the test–retest reliability and behavioral correlates of pupil responses in the n-back task

**DOI:** 10.1038/s41598-026-46731-3

**Published:** 2026-04-11

**Authors:** Gábor László Bényei, Péter Pajkossy

**Affiliations:** 1 Department of Cognitive Science, Faculty of Natural Sciences, Budapest University of Technology and Economics, Műegyetem Rkp. 3., Budapest, 1111 Hungary; 2 Neurocognitive Research Centre, Nyírő Gyula National Institute of Psychiatry and Addictology, Lehel U. 59., Budapest, 1135 Hungary; 3 Cognitive Medicine Research Group, Competence Centre for Neurocybernetics of the Life Sciences Cluster of the Centre of Excellence for Interdisciplinary Research, Development and Innovation of the University of Szeged, University of Szeged, Szeged, Hungary

**Keywords:** Pupil size, Pupillometry, Individual differences, n-back, Test–retest reliability, Working memory, Attention, Neuroscience, Psychology, Psychology

## Abstract

Measurement of pupil size changes is a promising method to investigate information processing. Our study contributes to this stream of research (1) by investigating whether pupillary changes during performing a demanding working memory (n-back) task are associated with performance, and (2) by testing whether these pupil responses demonstrate temporal stability over a period of multiple weeks. We conducted two experiments using two different variants of an n-back task differing in task durations and found that both phasic and tonic pupil size measures are characterized by good test–retest reliability. Furthermore, phasic, task-evoked pupillary responses were positively correlated with task performance, whereas no evidence was found for a similar link in the case of tonic pupil size measures, possibly due to design characteristics. Importantly, the dissociation of validity and reliability regarding tonic pupil size measures suggests that pupil responses might be temporally stable also in such cases when the pupil response itself is not predictive of task performance. Our results imply that pupillometry can be an appropriate tool to assess individual differences in cognition or underlying neural functioning, but one must ensure that the between-sessions correlations among participants are caused by individual differences in information processing, and not by other methodological factors (e.g. participant-specific, systematic measurement noise).

**Supplementary Information:**

The online version contains supplementary material available at 10.1038/s41598-026-46731-3.

## Introduction

As decades of research demonstrate, pupil size changes might mirror activity of several subcortical nuclei, related functional networks, and neurotransmitter systems (for reviews, see^1–5^). Because of this link between brain networks and pupil size, pupillometry can be considered as an efficient and promising way of investigating how specific brain processes underlie information processing.

The vast majority of studies examining this link focused on transient, 1–2 second long pupil dilations, which can be observed in response to task-relevant stimuli, as a marker of increased processing demands (for reviews see^3,6,7^). These task-evoked pupil responses (TEPRs) are about 0.1–0.5 mm large, and are evoked by processing related to a variety of different cognitive functions. Importantly, their magnitude increases with higher task difficulty (e.g.^8,9^), and thus they are thought to signal the amount of attentional resources or mental effort to solve a specific task^6^. The absolute size of the pupil, relative to which these pupil dilations emerge (i.e. baseline pupil size), is also changing constantly, and its variation is suggested to reflect changes in the underlying attentional and motivational state of the organism^10–14^.

The dichotomy between transient pupil responses and more slowly evolving baseline variations parallels the distinction between phasic and tonic changes in the firing patterns of the locus coeruleus (LC), a small subcortical nucleus responsible for the vast majority of noradrenergic (NA) neurotransmission in our brain^10^. As LC firing patterns are reflected in pupil responses^1,15,16^, the link between pupil size changes and information processing can be interpreted in the framework of the adaptive gain theory^10^, which describes how NA projections originating from the LC (called the LC/NA system) regulate attention, complex behavior and higher-level cognitive processes.

The adaptive gain theory suggests that NA modulation of attention and action planning operates in a bistable manner, causing the agent to exploit available resources until the expected utility of the behavior drops below a certain threshold—in this case, the agent rapidly shifts to an explorative state, promoting behaviors seeking for new opportunities and higher rewards. According to the theory, both tonic firing level and phasic burst-like activity of the LC signal changes in the arousal state, also affecting pupil diameter. On the one hand, task-related phasic bursts of LC firing are thought to optimise task-relevant processing in the involved cortical networks, and this process is then reflected in TEPRs. On the other hand, long-term alterations in tonic firing rate of the LC and consequently in baseline pupil size are linked to changes in task engagement (e.g.^10,12^) or behavioral strategies (i.e. shifting between exploitation and exploration, see e.g.^17–20^). Specifically, small baseline pupil diameter reflecting low tonic LC firing rate is associated with low engagement and poor task performance, whereas a large baseline pupil size mirroring high LC firing rate is observable when participants are distractible or they are exploring the environment without a clear behavioral goal (see e.g.^21–23^). Consequently, medium levels of baseline pupil size reflecting intermediate tonic LC firing rate are linked to optimal task engagement and attentional focus. Moreover, task-related phasic LC activity is also the largest for this intermediate tonic activity.

These links between pupil size changes, cognitive functions and neural processing open up the possibility to use pupillometry in applied settings, for example, to screen for signs of cognitive decline in elderly participants or to assess cognitive ability in educational settings. To use pupillometry for these purposes, one should show that individual differences in pupil size metrics reflect individual variation in controlled attention (i.e. the question of validity) and that pupil size metrics reveal trait-like characteristics of a given individual with temporal stability (i.e. the question of test–retest reliability). In the following, we summarize research on both of these aspects.

### Individual differences in pupillary responses reflect variation in controlled attention—the question of validity

Adaptive behavior necessitates controlled attention, that is, the ability to focus on the task-relevant information, while monitoring the surroundings and inhibiting the processing of task-irrelevant stimuli^24–26^. Several influential theories suggest that controlled attention is a key concept when explaining the ability of our brain to actively maintain and manipulate task-relevant representations, a function termed working memory^27–29^. The studies mentioned in the previous section (see e.g.^9,23^) suggest that pupil size changes measured for a given individual may reflect brain processes determining attentional focus, task engagement, and working memory performance. Importantly however, persons may vary in the degree of attentional effort they are capable of or willing to pay to the task at hand^30,31^. From this follows that individual differences in attentional control might be predicted by individual variation in pupil size measures. Proving such a link would facilitate the use of pupillometry for investigating differences in attentional control in both normal and clinical populations. Importantly, based on the adaptive gain theory, between-subjects differences should be detected both for the magnitude of the task-evoked pupil responses and also for the variability of baseline pupil size at a trial-by-trial level.

Regarding task-evoked pupil responses, two contrasting hypotheses can be put forward: According to the effort account^32^, we would expect that better performance in a task requiring controlled attention should be associated with greater TEPRs, as high-performers can recruit a larger/more active cortical network through increased LC phasic firing. Conversely, according to the efficiency account, we could hypothesize that better performers would show smaller TEPRs^33^ because they can process information more efficiently, with less facilitation of cortical activation through the LC/NA system. Results from previous research are more aligned with the former account: participants who tend to show better performance in attentionally demanding tasks typically produce larger transient pupil responses, while the results depend on whether the task is generally harder or easier for participants to perform^32,34,35^.

Regarding slower, tonic pupillary diameter changes, existing experimental evidence supports the notion that individuals who exhibit lower variability in their pre-trial baseline pupil size show better task performance^35,36^, as they might be able to maintain a constant level of optimal arousal throughout the task, that is they can minimise off-task periods and mind-wandering (see also^37,38^).

These results are in line with the *intensity-consistency framework*, proposed recently by Unsworth and Miller^39^. According to the authors, individuals who are able to exert attentional effort more intensively and consistently, are expected to exhibit better cognitive performance. This account is congruent with findings stating that larger TEPRs (intensity) and smaller variation in pre-trial baseline pupil size (consistency) predict better task performance (see^32,34–36^).

### Temporal stability of pupil size measures—the question of reliability

Whereas results presented in the previous section suggest that pupil size changes can be valid measures of individual differences in task-related attentional allocation, the applicability of pupillometry for assessing such differences also depends on the temporal stability or test–retest reliability of pupil size measures. Specifically, if we assume that pupil responses are informative of temporally stable characteristics of cognitive ability, then these pupil responses should be characterized by temporal stability.

To the best of our knowledge, there is only a limited number of studies investigating the test–retest reliability of pupil size measures, mostly originating from clinical assessment literature. In these studies, test–retest reliability of task-evoked peak pupil dilation, as well as peak latency, was found to be generally good, between occasions ranging from hours to weeks apart^40–43^.

Importantly, pupil size and dilation metrics are also determined by measurement noise: in most applications, size of the pupil is measured using the video-images of an infrared camera: the recorded images are processed using image processing and feature detection algorithms specific to the eye-tracker software, which detects the pupil(s) on the image and estimates their diameter. This process is very sensitive to distortions: low spatial and/or temporal resolution of relevant portions of the image stream, inhomogeneous illumination, fluctuations in camera-to-head distance, head movements, low-confidence identification of the pupil contour, and artefacts related to blinks and saccades^44–50^. These factors are difficult to control for, and the measurement noise they cause is mostly inherent in the data researchers receive from the eye-tracker. Crucially, some of these factors might be related to the participant (e.g. head movement frequency, anatomical characteristics of the eye and eyelid). Thus, test–retest correlations might partly be the result of such participant-specific factors, which are independent from individual differences in attentional control. In order to tease apart these two, in our study, we aimed to test both the validity and the test–retest reliability of pupil responses, as measures of between-subjects differences in attentional allocation and control.

### Testing validity and temporal stability of pupil responses using an n-back task—the current study

As summarized previously, pupil responses during demanding cognitive tasks are suggested to reflect individual differences in attentional allocation, due to the modulatory involvement of LC in attentional regulation^31,34,35,38,51^. The link between individual performance and pupil size changes might reflect between-subjects differences in how intensely and consistently participants are paying attention to the given task^36,38,52,53^. Whereas several studies suggest that pupil size measures are valid^32,34–36,54^ and temporally stable^40,42,43^ indices of attentional allocation, we set out to replicate and complement these results by the simultaneous investigation of both aspects. Specifically, we inspected both the strength and the stability of the link between pupillary changes and individual differences in attentional performance over a time period of weeks, using a visual n-back paradigm (For similar paradigms, see e.g.^34,36^).

In the n-back task^55^, a sequence of stimuli is presented, one at a time, and participants are instructed to encode each stimulus, compare it with the one seen a specific number of items before, in order to indicate matching cases. By increasing the lag (entitled n), task difficulty rises as participants have to maintain a larger set of recent elements while they constantly need to update this set at every new stimulus. Many variants of the task exist depending on whether the stimuli are visual or auditory, whether they carry perceptual or semantic content, whether only target trials or all trials require an active response, and most importantly, what the n parameter is^56,57^.

We opted to employ an n-back task because it is a widely used task assessing how participants can update and control the contents of their working memory. It is an attentionally demanding task where individual differences are expected to emerge, and the 2-back version is easy enough to be solved by our healthy young adult participants, though it is challenging enough to produce variance in individual scores, which is necessary for correlational analyses. Furthermore, several previous studies applying the n-back paradigm have already found evidence that individual differences in pupil size measures are related to task performance^34,36,54,58^.

We used a visual 2-back variant of the task by presenting single digits on the screen, and participants had to indicate whether the current stimulus was the same as the one presented two items before. To measure task performance, we calculated the sensitivity (*d*_L_) measure, which is a composite score combining hit rate and false alarm rate^56,59,60^, and calculated several pupil size metrics, which were previously found to be linked to task performance: the measures consisted both indices of phasic (magnitude of TEPR, see e.g.^34,35^) and tonic pupillary activity (baseline pupil size variability^23,35,36,38,53,61^).

Furthermore, Robison and Garner^36^ reported that not only the variability, but also the mean of pre-trial baseline pupil size values were positively correlated with n-back performance—this pattern might be explained by a somewhat controversial set of results in the literature, suggesting that individual differences in absolute pupil size are related to individual differences in working memory performance or fluid intelligence (e.g.^62,63^). Other investigators failed to observe this correlation^51,64^ (for a meta-analysis see:^65^), thus, to add further observations regarding the existence of this link, we also investigated the validity and reliability of mean pre-trial baseline pupil size. We aimed to test four specific research questions.

First, we tested whether the magnitude of TEPRs, as a proxy measure of the intensity of attentional allocation (e.g.^35^), correlates with task performance across participants in both sessions, separately. Two measures were calculated for each individual: TEPR averaged for target hit trials (i.e. when participants successfully detected a 2-back case), and for correct rejections (CRs; i.e. when participants correctly indicated that there was no 2-back case). Greater pupillary response to both target hits and CRs was expected to predict better performance (see e.g.^34^). Second, we also investigated whether the variability of pre-trial baseline pupil size, as a correlate of the consistency of attentional allocation, predicts task performance. Specifically, in accordance with previous findings^23,35,38,53^ (see also^36,61^), we expected that higher pre-trial variability would be associated with lower performance. Third, we aimed to replicate the positive correlation between the mean pre-trial pupil size and n-back task performance, as shown by Robison and Garner^36^. Fourth, to investigate test–retest reliability of task performance and pupil responses, we calculated the correlation of the behavioral and pupillary metrics between the two sessions of the same task completed by the same participants. Based on previous findings showing moderate to good test–retest reliability of accuracy in the n-back task^66,67^, we expected a positive correlation of the same sensitivity (*d*_L_) measure between the two sessions. Furthermore, we expected good temporal stability of pupil size measures^40–43,68^. Finally, we also combined our within-session and between-sessions analysis approach, and used mediation analyses to investigate the shared and independent variance in the temporal stability of task performance and pupil size metrics.

Our research aim was to investigate how pupillometry could be applied as an easy-to-use methodology to assess individual differences in the neural processes underlying attentional control. Thus, in our first study, we chose to test the validity and reliability of a rather short version of the n-back task (task duration around five minutes), which can be realistically assumed to be a tolerable task duration even in clinical settings. This short version was administered in two subsequent sessions with a delay of multiple weeks, and we measured TEPRs evoked by information processing during the task. As described below, the results of this first study only partially confirmed our predictions—thus, we conducted a second study, where task duration was increased significantly (around 20 minutes). Due to the resulting increase in statistical power, the pattern of results in this second study was more in line with our predictions.

## Experiment 1—using a short version of the n-back task

### Methods

#### Participants

One of our main research aims was to show the correlation between pupil size measures and n-back performance, thus we calibrated our sample size to show this effect. At the start of our study, we were aware of only one study investigating this issue^34^, thus we could not estimate a reliable population effect size from previous results. Because of this, when performing power analysis to justify sample size, we assumed that the magnitude of the effect in the population is relatively low, but still meaningful, and thus used an a priori effect size estimation of *r* = 0.4. We used GPower^69^ to compute the required sample size to detect this effect, which resulted in a required sample size of 61, by setting the statistical power to 0.90. To compensate for potential data loss, we recruited slightly more participants than suggested by the power analysis. Data were collected from 75 university students (48 female, *M*_age_ = 21.4 years, *SD*_age_ = 1.9, *Range* = 18–27). All participants were given monetary compensation after completing the two sessions. These occasions were within a few weeks temporal distance, with some variation due to various reasons related to the organisation of the data collection (*M*_dist_ = 19.9 days, *SD* = 7.6, *Range* = 6–39). Participants were asked in advance to restrict drinking beverages containing caffeine within six hours and smoking within two hours of the start of the experimental sessions. In the debriefing we asked whether they adhered to these restrictions, and all of them indicated yes. They were informed about all relevant aspects of the experiment, and they gave written consent to participate. The research project was carried out in accordance with the Declaration of Helsinki, as approved by the United Ethical Review Committee for Research in Psychology, Hungary.

For within-session analyses of individual pupillary and behavioral metrics, we excluded participants based on their behavioral performance and eye recording data quality. Exclusion of participants was performed in three stages. In the first stage, we excluded participants who allegedly misunderstood the instructions or were unmotivated to perform the given task. We did this by disregarding those who did not give a required behavioral response in at least 50% of all trials, or produced false alarms (incorrectly indicated a target) in case of at least 50% of all trials (session 1: six participants excluded; session 2: eight participants excluded). The next stage was made to exclude participants whose eye data was not reliable because their percentage of mean interpolated time length within trials laid more than three standard deviations away from their group average (session 1: three participants excluded; session 2: one participant excluded, see details later). This percentage refers to the amount of missing data (due to blinks) during the time between stimulus onset and offset in each trial, averaged across all trials for an individual in a given session, multiplied by 100 to yield a percentage of how much of the average trial length was unknown and had to be interpolated by the surrounding samples in the eye recording. In the third stage, we excluded participants, if any of their pupil-derived metrics were found to be outliers: specifically, we excluded participants, if their pre-trial baseline pupil size variability, or average TEPR value for either hits or CRs, respectively, laid more than three standard deviations away from their group average. Due to this, we had to exclude one participant from session 1 and one participant from session 2. Also, apart from the mentioned exclusions, data of one participant was unusable from the first session due to a recording error, and one additional recording was lost from the second session.

After these exclusions, for within-session analyses, the ultimate sample size was N = 64 for session 1 (38 female, *M*_age_ = 21.5 years, *SD*_age_ = 2.0, *Range* = 18–27), and N = 63 for session 2 (39 female, *M*_age_ = 21.4 years, *SD*_age_ = 2.0, *Range* = 18–27). For between-sessions analyses of individual pupil measurements and behavioral performance metrics, we excluded participants listwise, meaning that a participant was excluded if their data were excluded for any of the two sessions. This exclusion resulted in a sample size of N = 56 for between-sessions analyses (34 female, *M*_age_ = 21.4 years, *SD*_age_ = 2.1, *Range* = 18–27).

#### Design and procedure

Participants were individually tested in a dimly lit room, isolated from any natural light. All equipment in the lab and screen brightness of the monitor were kept constant during all data recordings. We checked the room from the participant’s eye facing the monitor with a portable digital LUX meter (Voltcraft LX-10) which resulted in a stable 14 LUX reading three consecutive times. Pupil size was registered with an SMI Red 500 type remote eye-tracker at 500 Hz^70^ running iView X software^71^ version 2.7.13. Gaze calibration was performed for each individual before starting the n-back task using a 5-point calibration and validation routine. The 2-back task was presented using PsychoPy 2021.1.4^72^ on a 60 Hz Dell monitor, while participants sat comfortably with their heads positioned at 58 cm viewing distance using a chin rest. Data processing was performed with MATLAB R2021b^73^ using our own written scripts. Sensitivity (*d*_L_) and bias (*c*_L_) scores were calculated in MS Excel^74^. We carried out classic correlational tests with JASP version 0.18.2^75^, and time-course correlation analyses with MATLAB, while intraclass correlation and mediation analyses were performed using SPSS^76^. For data visualization, we used MATLAB and RStudio version 2023.09.0^77^.

The experiment employed a visual 2-back paradigm with numeric stimuli (digits from one to nine), presented on 50% grey background with white font colour, all within 1.5 degrees of viewing angle to the foveated screen center. Participants were instructed to respond to each perceived stimulus by pressing the up or down arrow keys on the computer keyboard, the former indicating target trials, and the latter indicating non-targets. That is, if participants thought that the currently presented stimulus was the same as the one presented two trials before (i.e. a 2-back trial), the up arrow key had to be pressed, and in all other cases, the down arrow key. Before the start of the experiment, participants were asked to confirm that they understood the instructions and were ready to perform the task. A total of 100 trials were presented sequentially with no break, which took a total of 5.3 min. Each trial was 3200 ms long, starting with a blank screen containing a fixation cross for 700 ms, followed by presentation of the actual stimulus, which remained visible for 2500 ms. Keypresses did not cause the presented stimulus to visually change or disappear, but participants were informed in advance that their responses would always be recorded by the computer. We recorded and analysed the first keypress captured during the period a stimulus was visible on the screen. All participants used only one hand to press the response keys. They received the same series of pseudo-randomized stimuli. Thirteen trials were target 2-back trials. No 1-back lures were present (i.e. when a stimulus is identical to the one presented just before). Accordingly, for every trial after the third one, there was a need to update the contents of working memory (i.e. only the last three digits were to be held active in working memory). Consequently, due to different processing requirements, the first two trials were, in all cases, excluded from averaging the individual TEPRs.

#### Data preparation

Pupil size values in millimeter format from the left eye were analysed in MATLAB. Zero values and data points during blinks were removed, relying on the output of the built-in event detection system^78^ of our eye-tracker. Each recording was then interpolated using the “interp1” MATLAB function and its “pchip” (piecewise cubic Hermite-interpolation) method with equidistant query points at 50 Hz. Savitzky-Golay filter of fifth degree was applied using a 800 ms (40 samples) window length.

We calculated the percentage of missing data due to blinks, during the time between stimulus onset and offset in each trial, averaged across all trials for an individual in a given session, to determine how much of the average trial length was unknown and had to be interpolated. In each of the two sessions, we disregarded data from participants whose mean interpolation percentage was more than 3 standard deviations away from the average of those who were included after exclusion stage one (*M*_session1_ = 4.39%, *SD*_session1_ = 5.58; *M*_session2_ = 5.98%, *SD*_session2_ = 5.31).

To produce the task-evoked pupil response curves of each recording session of each individual, pupil data were broken down to epochs representing trials (each spanning from the start of the fixation cross to the end of the stimulus presentation). The number of trials used for TEPR and pre-trial baseline calculation per participant and per trial group can be found in the openly available data repository of this study. These trials were then baseline-corrected by subtracting the pupil size average of 200 ms before stimulus onset, and all trials belonging to the same condition of a participant were averaged to produce the mean pupil response curve (i.e. the TEPR) of the individual for that given condition (i.e. target hits and CRs).

After computing these two TEPR curves for all participants, they were used to measure the task-evoked peak pupil dilation for hits and CRs separately, by averaging the section between 1000–1500 ms related to stimulus onset. This time interval was determined by visually inspecting the grand average TEPR curves for hits and CRs for all participants (see results section). These peak TEPR values were used in our subsequent analyses to assess the magnitude of the pupil responses.

We also analysed metrics derived from variation in pre-trial baseline pupil diameter between trials. Specifically, baseline of each trial was calculated as the average of 200 ms preceding stimulus presentation. Then, the coefficient of variation between these pre-trial baseline pupil size mean values was calculated for each participant separately. This was done by dividing the standard deviation of these pre-trial mean values by their mean across trials. This mean value was used to assess mean pre-trial baseline pupil size of the participants. Importantly, we only included baseline values that corresponded to trials not biased by an error (false alarm/miss) or a target hit from the preceding trial. Due to differences in cognitive processing requirements, we also excluded data from the first two trials during the calculation of both the TEPR values and pre-trial baseline pupil diameter (see above). Descriptive statistics of the different pupil size metrics for both sessions are presented in the Supplementary Material, in Table S1.

Behavioral performance in each session was described using the sensitivity (*d*_L_) and bias (*C*_L_) metrics, computed using the formulas proposed by Snodgrass and Corwin^60^ (see also^59^). According to the Signal Detection Theory^79^, sensitivity represents how well an agent can separate the signal from noise, and thus it is a frequently used measure to assess behavioral performance, whereas bias corresponds to how liberal the detection process is. In the context of the n-back task, sensitivity is practically a metric for the ability to distinguish between relevant stimuli (target 2-back trials) and irrelevant ones (non-target trials), while bias corresponds to the tendency of the participants to respond affirmatively in general^80^. To compute these metrics, hit rate and false alarm rate calculations were also necessary beforehand. Individual hit rates were calculated by dividing the number of hits by the total number of possible targets (i.e. by 13). Due to the specifics of sensitivity (*d*_L_) calculations, 0.0 values were recoded as 0.001, and 1.0 values were recoded as 0.999. Individual false alarm rates were calculated by dividing the number of hits by the total number of possible non-targets (i.e. by 85, excluding the first two trials as they needed no response), here again using the same recoding of 0.0 and 1.0 values. Note that sensitivity (*d*_L_) will be used in all subsequent analyses, but descriptive statistics of hit rate, false alarm rate, and bias are also presented to provide an overview of behavioral performance in each session.

#### Statistical analyses

Analyses regarding the link between pupil size measures and task performance were performed by computing correlations between sensitivity (*d*_L_) and the pupil size measures (baseline pupil size variability, mean, and peak TEPR values for hits and CRs). These correlations were computed separately for both sessions (i.e. within-session analyses). As several variables of pupil size measures and also sensitivity (*d*_L_) in the n-back task were non-normally distributed, to enable the comparison between different coefficient values, Spearman rank-based correlations were computed in all cases. Additionally, to investigate the temporal dynamics of possible correlations, time-course analyses were also conducted, using the averaged TEPR curves of the participants. That is, we computed a series of Spearman rank-based correlation coefficients between n-back sensitivity (*d*_L_) and baseline-corrected pupil dilation for each timepoint of the TEPR during the 2500 ms length of stimulus presentation (for similar approaches see e.g.^81–83^).

To test the reliability of pupillary responses, the correlation of pupil size measures between the two sessions was also assessed (between-sessions analyses). Again, due to non-normality issues and comparability of different results within the study, Spearman rank-based correlation coefficients were computed in all cases. Specifically, we calculated this correlation separately for peak TEPR values measured for target hits and CR trials. Similarly to the within-session analyses, we also carried out time-course correlation analyses between the two sessions, using the TEPR curves of different trial types. Specifically, each baseline-corrected timepoint of a specific TEPR curve in session 1 was correlated with its corresponding timepoint on session 2, across all participants (*N* = 56). We also assessed the reliability of pre-trial baseline pupil diameter variability and mean between task sessions by computing the correlation between the variability and mean values of the two sessions. Furthermore, as intraclass correlation coefficients (ICC) are more appropriate for determining the test–retest reliability of a measure, besides Spearman correlation coefficients, we also report ICC values with 95% confidence intervals. Specifically, to compute ICC, we use average-measurement, absolute-agreement, 2-way mixed-effects model. To interpret these ICC values, we will use the guidelines provided in^84^ (below 0.5: poor, 0.5–0.75: moderate, 0.75–09: good, above 0.9: excellent reliability).

**Fig. 1:**
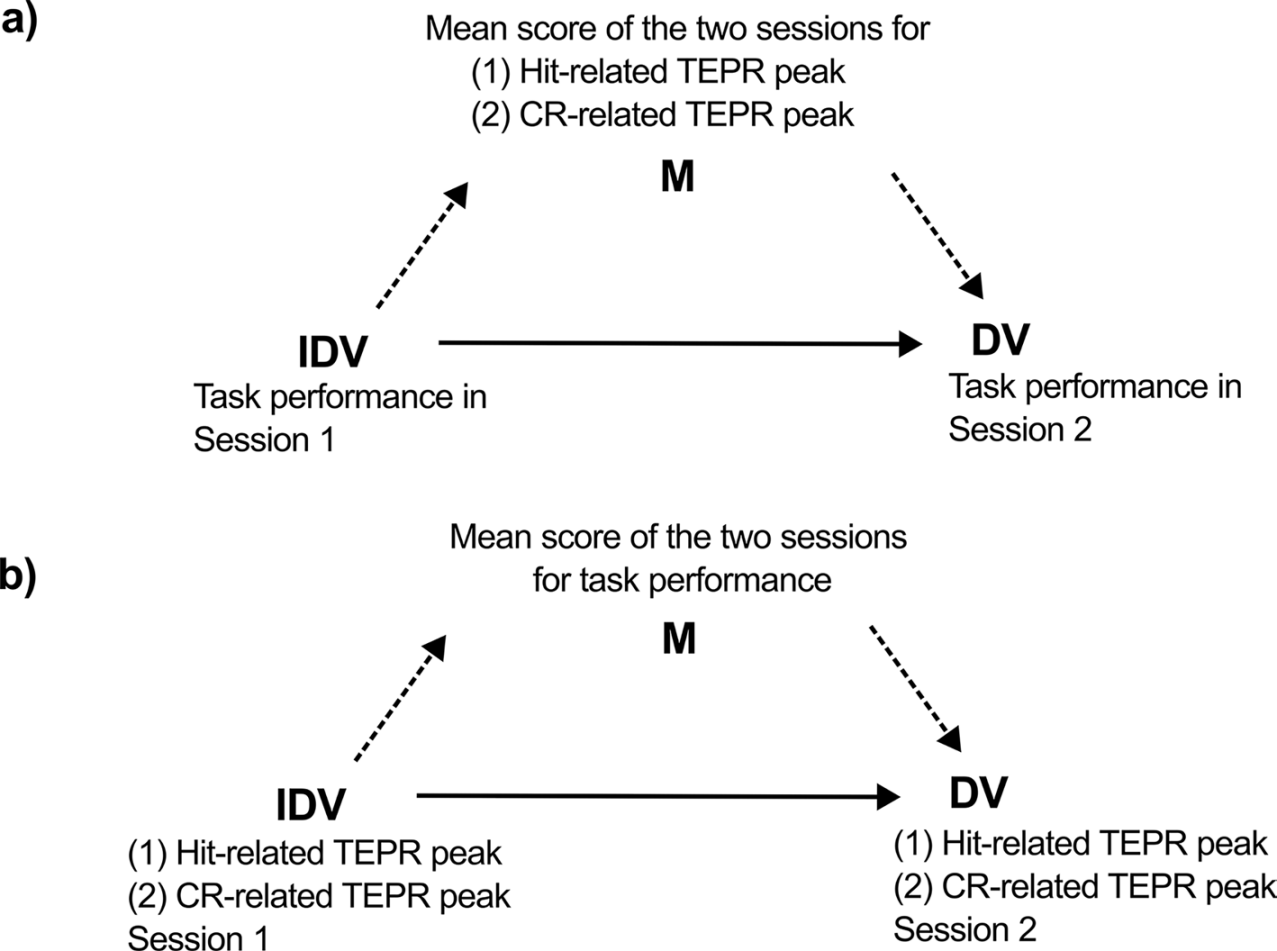
Mediation analyses. *Note:* We used two simple mediation models to investigate how between-sessions correlations of (**a**) task performance or (**b**) pupil size metrics**)** are mediated by mean scores of (**a**) pupil size metrics or (**b**) task performance, respectively. Both models were run separately for the two pupil size metrics. IDV: independent variable; DV: dependent variable; M: mediator variable.

Finally, we used mediation analysis to investigate the dependent and independent fractions of variance in the correlation between pupil size metrics and behavioral performance. To this aim, we first computed for each participant the mean of the two sensitivity (*d*_L_) values measured at the two sessions—this was taken as a composite measure of task performance. Similarly, mean values of the pupil indices were also computed (e.g. for each participant, we averaged the two values of hit-related TEPRs, measured during the two sessions). These mean values reflect the task performance and pupil responses of the participant, and are filtered for random measurement noise specific to one session (originating from e.g. fatigue or eye-tracking noise). Importantly, the measures of pre-trial baseline pupil size mean and variability were not involved in the mediation analyses, as the zero-order correlations between these pupil size metrics and task performance suggested no relationship (see later in results)—thus no mediation effects could be expected. After computing these mean values, a series of two simple mediation analyses was conducted to test whether the between-sessions correlation in task performance is mediated by their correlation with the mean score of the different pupil metrics (see Fig. 1a, dashed arrows). A significant mediation would prove that pupil size indices measure stable individual differences in task performance. In a second set of mediation analyses, we tested whether the correlations of the two pupil size metrics are mediated by the mean score of task performance. Here, we were interested whether we can find a significant direct effect (once the effect through the indirect path is controlled, see Fig. 1b, solid line): this would suggest that the correlation between the pupil size metrics has also non-cognitive sources that are independent from task performance (i.e. which is related to participant specific measurement error). We used the PROCESS macro in SPSS (version 4.3^85^), which is conceptually similar to the classic Sobel test^86^ frequently used in mediation analysis, but computes robust bootstrapped parameter estimates for the indirect effect.

### Results

The descriptive statistics of relevant variables are summarized in Table 1. As can be seen from the hit rate and false alarm rate measures, participants had no difficulties solving the task. Also importantly, data do not indicate ceiling effects, because performance was far from perfect.

**Table 1:** Descriptive statistics.

Metric	Session 1						Session 2					
	Hit rate	FA rate	Sensitivity (*d*_L_)	Bias (*c*_L_)	Hits RT (ms)	CRs RT (ms)	Hit rate	FA rate	Sensitivity (*d*_L_)	Bias (*c*_L_)	Hits RT (ms)	CRs RT (ms)
N	64						63					
M	0.830	0.058	5.912	0.542	969	1107	0.823	0.045	6.085	0.660	862	965
SD	0.152	0.067	2.936	1.128	198	261	0.164	0.056	3.025	0.991	215	274
Min	0.308	0.001	1.204	− 2.598	608	547	0.154	0.001	0.974	− 2.387	442	397
Max	0.999	0.318	13.814	2.851	1482	1674	0.999	0.306	13.814	2.851	1720	1855

Reaction times are in milliseconds. Individual hit rates were calculated by dividing the number of hits by the total number of possible targets (13), limited at 0.001 from below and at 0.999 from above. Individual false alarm rates were calculated by dividing the number of hits by the total number of possible non-targets (85, excluding the first two trials as they needed no response), limited at 0.001 from below and at 0.999 from above. FA: False alarm; CRs: Correct rejections; RT: Response time.

Participants’ reaction times improved significantly to the second session for both hits (t(55) = 3.722, *p* < 0.001, d = 0.497) and CRs (t(55) = 4.887, *p* < 0.001, d = 0.653), indicating a practice effect, although such effect was not observed for their sensitivity (*d*_L_) scores (t(55) = -0.719, *p* = 0.475, d = -0.096).

#### The link between pupillary measures and task performance—the question of validity

##### Session 1

**Fig. 2:**
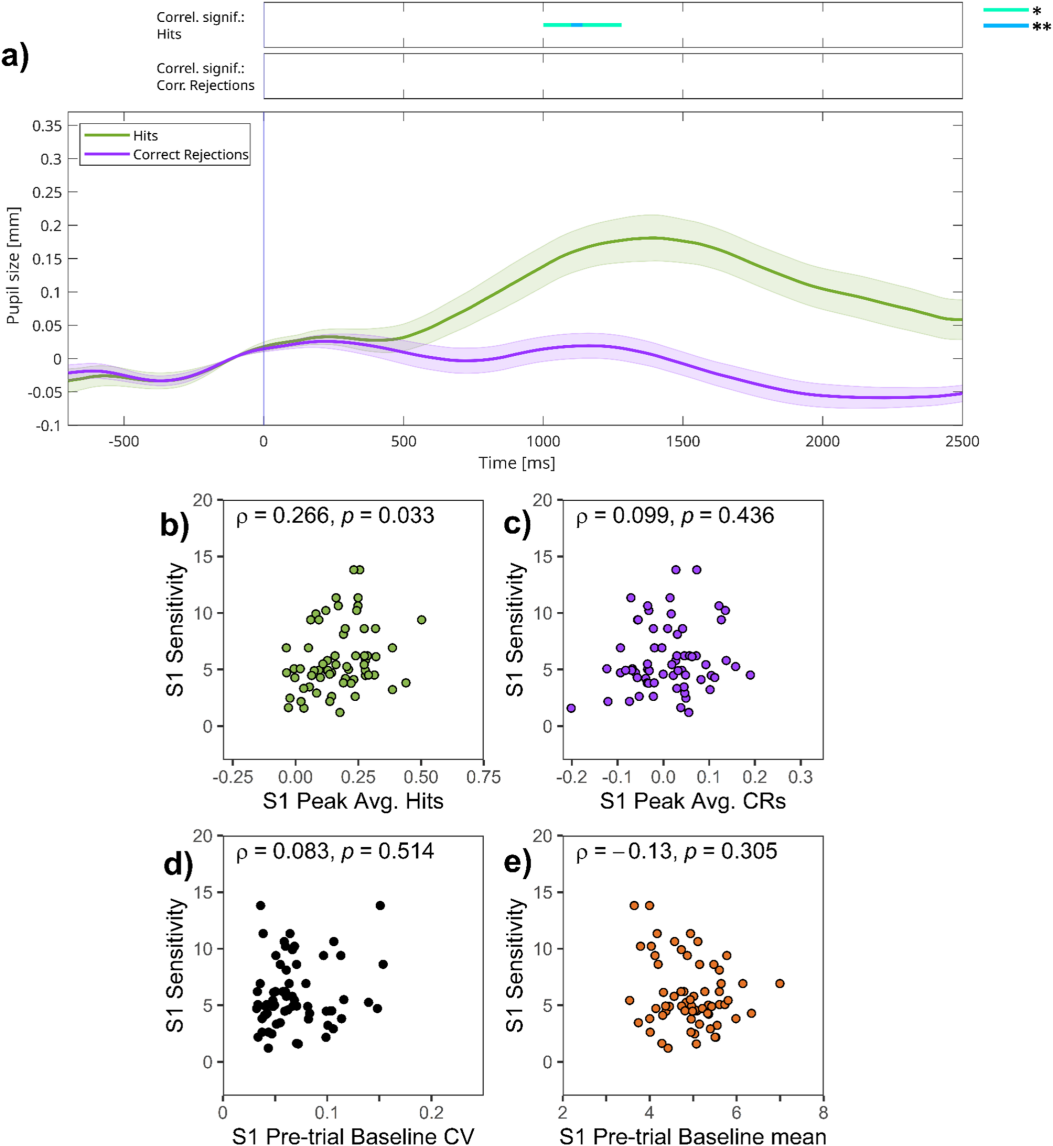
Session 1 of experiment 1—(**a**) Task-evoked pupillary responses and (**b**–**e**) correlations of pupillary responses and task performance. *Note:* (**a**) Grand average task-evoked pupillary response (TEPR) curves for target hits and correct rejection (CR) trials. Pupil size curves are stimulus-aligned, baseline-corrected for the preceding 200 ms, and averaged across all trials and participants. The bars on the top represent regions of significance, regarding time-course analyses, correlating the event-related pupil response curves and sensitivity (*d*_L_) scores in session 1 (*N* = 64). Pupil size metrics are in millimeters. Colored areas represent 95% confidence intervals. (**b**–**e**): Scatter plots showing the link between sensitivity (*d*_L_) and: (**b**) TEPR peak values for target hits, (**c**) TEPR peak values for CRs, (**d**) pre-trial baseline pupil size variability (CV: coefficient of variation), (**e**) pre-trial baseline pupil size mean.

TEPR curves for hits and CRs in session 1 are presented in Fig. 2a. As can be seen, hits evoked a clear pupil dilation 1–2 s after the presentation of the stimulus (green line on Fig. 2a), whereas pupil responses for CRs are smaller (purple line on Fig. 2a). First, we assessed the correlation between sensitivity (*d*_L_) and TEPR peak values, calculated as an average of the 1000–1500 ms interval after stimulus presentation. In contrast to our hypotheses, sensitivity (*d*_L_) was related significantly to TEPR peak values either for target hits (ρ = 0.266, *p* = 0.033; Fig. 2b) but was not for CRs (ρ = 0.099,* p* = 0.436; Fig. 2c). Despite these non-significant correlations, we investigated how sensitivity (*d*_L_) correlates with the magnitude of pupil dilations at each time point of the stimulus presentation period. To this aim, time-course analysis was conducted (indicated by top bars on Fig. 2a, see Methods section for details; see also Figure S1 in the Supplementary Material). As can be seen, only small periods of the trial duration were characterized by significant correlation between sensitivity (*d*_L_) and moment-to-moment pupil size changes. This is in line with the results of our analysis using TEPR peak values.

Second, we also investigated the link between pre-trial baseline pupil size, its variability and task performance, and contrary to our hypothesis, we did not find a correlation between pre-trial baseline variability and n-back sensitivity (ρ = 0.083, *p* = 0.514; Fig. 2d), and did not find a correlative relationship with baseline mean (ρ = -0.130, *p* = 0.305; Fig. 2e) either.

To sum up, we could demonstrate the predicted positive correlation between TEPR peak for target hits and sensitivity (*d*_L_), but we did not find a significant relationship between pre-trial baseline pupil size and task performance.

##### Session 2

**Fig. 3:**
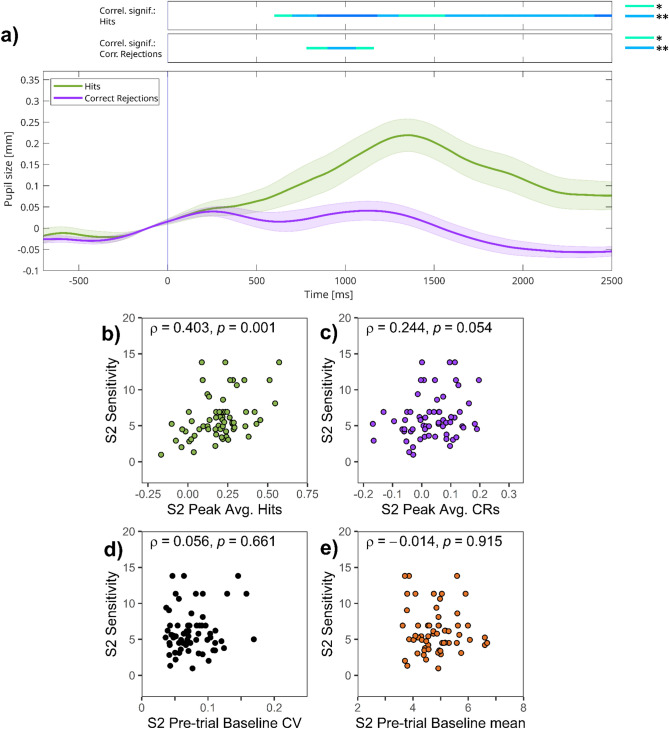
Session 2 of experiment 1—(**a**) Task-evoked pupillary responses and (**b**–**e**) correlations of pupillary responses and task performance. *Note:* (**a**) Grand average task-evoked pupillary response (TEPR) curves for target hits and correct rejection (CR) trials. Pupil size curves are stimulus-aligned, baseline-corrected for the preceding 200 ms, and averaged across all trials and participants. The bars on the top represent regions of significance, regarding time-course analyses, correlating the event-related pupil response curves and sensitivity (*d*_L_) scores in session 2 (*N* = 63). Pupil size metrics are in millimeters. Colored areas represent 95% confidence intervals. (**b**–**e**): Scatter plots showing the link between sensitivity and: (**b**) TEPR peak (1000–1500 ms average after stimulus onset) values for target hits, (**c**) TEPR peak (1000–1500 ms average after stimulus onset) values for CRs, (**d**) pre-trial baseline pupil size variability (CV: Coefficient of variation), (**e**) pre-trial baseline pupil size mean.

The shape of TEPRs (Fig. 3a) is similar to what was found in session 1. We first assessed the correlation between sensitivity (*d*_L_) and TEPR peaks, calculated as an average of the 1000–1500 ms interval after stimulus presentation. Peak pupil size for target hits correlated with sensitivity (ρ = 0.403, *p* = 0.001; Fig. 3b), but peak size for CRs did not (ρ = 0.244, *p* = 0.054; Fig. 3c). These correlations were also examined on a moment-to-moment basis, using time-course analyses (bars on top of Fig. 3a; see also Figure S2 in the Supplementary Material). As can be seen, the positive and significant correlation between TEPR evoked by hits and task performance emerges after 500 ms, and can be consistently found for the whole duration of the stimulus presentation period. For TEPRs evoked by CRs, the link between moment-to-moment pupil size changes and sensitivity (*d*_L_) was only significant for a small time section between 800 and 1200 ms.

Second, we also investigated the link between pre-trial baseline pupil size, its variability and task performance, and found no significant correlation between them (ρ = 0.056, *p* = 0.661; Fig. 3d), and neither for baseline mean (ρ = -0.014, *p* = 0.915; Fig. 3e).

Thus, in session 2, TEPRs evoked by target hits were correlated with task performance, whereas we failed to find a significant correlation between sensitivity (*d*_L_) and either TEPRs evoked by CRs or by baseline pupil size variability.

#### Correlation of pupillary responses between two sessions—the question of test–retest reliability

**Fig. 4:**
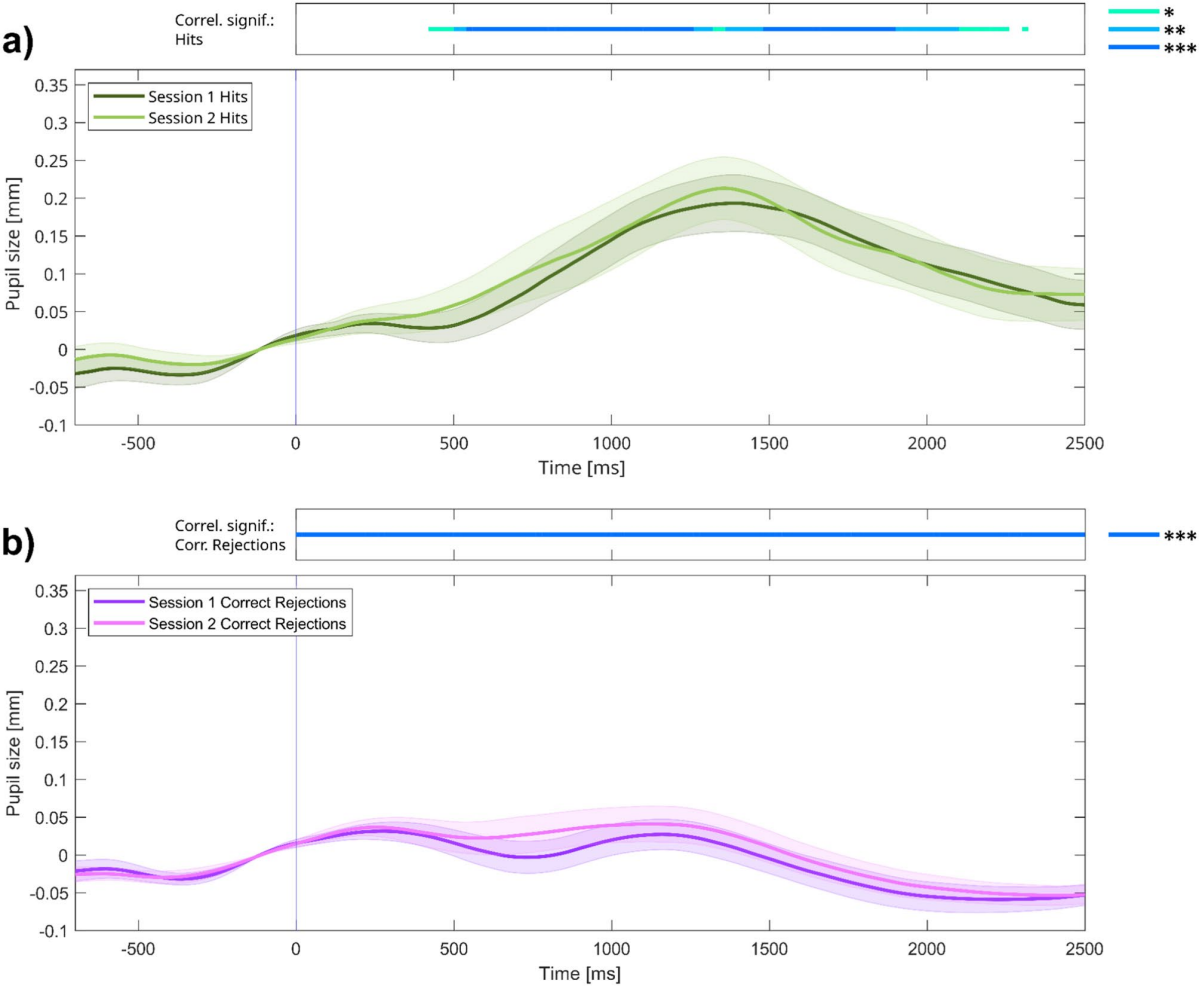
Task-evoked pupil responses of sessions 1 and 2 in experiment 1, for (**a**) hits and (**b**) correct rejections (CRs). *Note:* (**a-b**): Grand average task-evoked pupillary response (TEPR) curves for target hit and CR trials, depicted for both sessions. Pupil size curves are stimulus-aligned, baseline-corrected for the preceding 200 ms, and averaged across all trials and participants. The bars on the top represent regions of significance, regarding time-course analyses, correlating the pupil size curves at the two sessions (*N* = 56). Colored areas represent 95% confidence intervals.

In Fig. 4a,b, we plotted TEPRs for hits and CRs, respectively, superimposed for the first and the second sessions. As can be seen on the graphs, the responses observed in session 1 and session 2 are quite similar, with respect to both peak magnitude and latency.

**Fig. 5:**
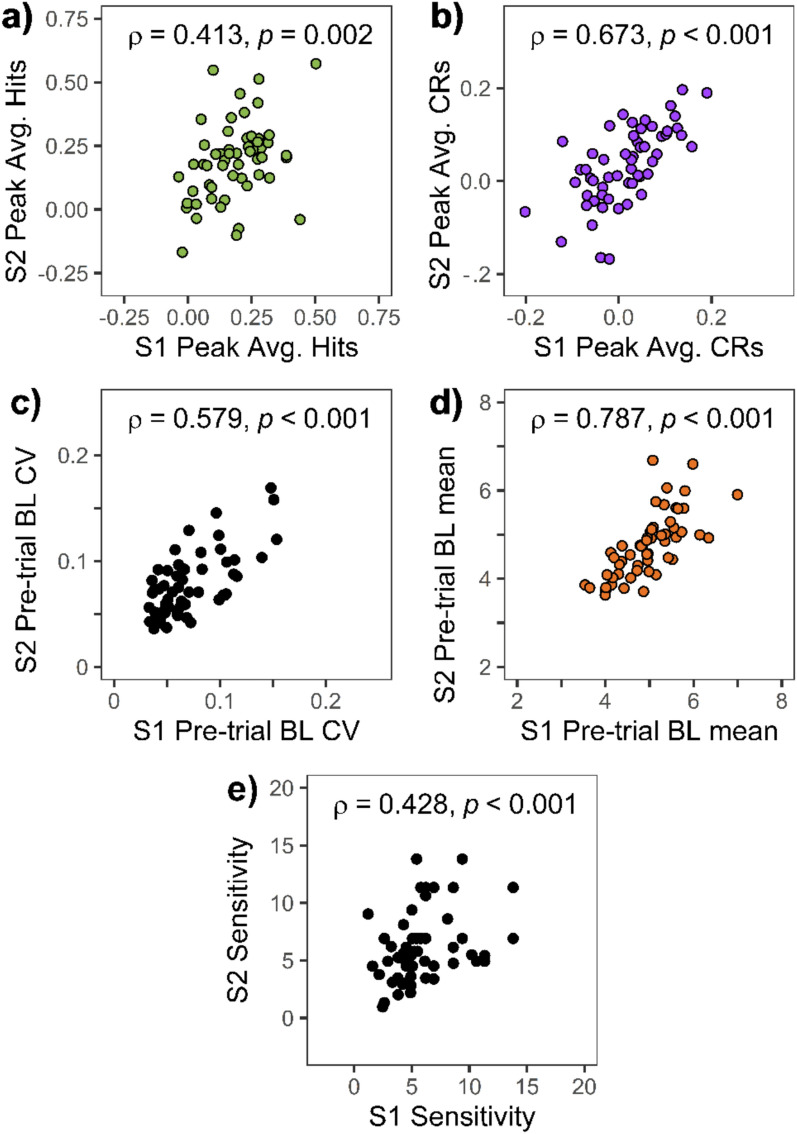
Between-sessions correlations of (**a**–**d**) pupil responses and (**e**) task performance. *Note:* Scatter plots showing the correlation between the values measured at the two sessions of experiment 1: TEPR peaks (1000–1500 ms average after stimulus onset) during (**a**) target hits and (**b**) correct rejections. Pre-trial baseline (BL) pupil size (**c**) variability (CV: Coefficient of variation) and (**d**) mean. (**e**) Sensitivity (*d*_L_) values. Pupil size metrics are in millimeters. (*N* = 56).

First, we investigated the correlation of TEPR peak values of the two sessions. This analysis revealed that TEPR peak values for target hits in session 1 predicted the same pupil responses in session 2 (ρ = 0.413,* p* = 0.002; *ICC* = 0.555, 95% CI: 0.237–0.740; Fig. 5a). A noticeably higher degree of correlation can be seen for the between-subjects correlation of TEPR peak values for CR trials (ρ = 0.673, *p* < 0.001; *ICC* = 0.778, 95% CI: 0.618–0.870; Fig. 5b). Using time-course analyses, we investigated the between-sessions correlations of individual TEPR curves on a moment-to-moment basis (bars on top of Fig. 4a,b; see also Figure S3 in the Supplementary Material). These analyses confirmed the results of the TEPR peak-based analysis, showing significant correlations for hits and CRs for the whole stimulus presentation period. Similarly to TEPR peak values, pre-trial baseline pupil size variability also exhibited temporal stability, as evidenced by a significant Spearman’s correlation (ρ = 0.579, *p* < 0.001; *ICC* = 0.802, 95% CI: 0.662–0.884; Fig. 5c), which was also true for baseline mean values (ρ = 0.787, *p* < 0.001; *ICC* = 0.813, 95% CI: 0.656–0.895; Fig. 5d). Importantly, sensitivity (*d*_L_) of behavioral responses also correlated between sessions (ρ = 0.428, *p* < 0.001; *ICC* = 0.541, 95% CI: 0.216–0.731; Fig. 5e). Thus, we could find that all four pupil size measures showed temporal stability over a period of multiple weeks, and the time-course analysis also suggests that this between-sessions correlation is present for the whole duration of the trial.

#### Mediation analyses

As the zero-order correlations between task-performance and baseline pupil size mean and variability were negligable in both sessions, only the TEPR metrics were involved in the mediation analyses. First, we investigated the correlation between the mean scores of task performance and pupil size metrics (averaged over the two sessions). We found that the mean score of sensitivity (*d*_L_) correlated significantly with the mean scores of both pupil metrics (hit-related TEPR: ρ = 0.444, *p* = 0.001; CR-related TEPR: ρ = 0.287, *p* = 0.032). Interestingly, whereas the correlation between pupil size metrics and task performance was not significant in all cases when investigating data from the two sessions separately, the correlations observed for the mean scores were significant and in the predicted direction for each of the two metrics, when investigating the composite data of the two sessions. This suggests that averaging over the two sessions attenuates measurement noise specific to the different sessions.

As a next step, we used mediation analysis to investigate whether the mean score of the two pupil size metrics (averaged over the two sessions) mediates the correlation between task performance in the two sessions (see Fig. 1a and the results in Table 2). Using mediation analysis, we estimated the extent to which the link of an independent variable to a dependent variable is explained by a third mediator variable (indirect effect) and to what extent it is independent of the mediator (direct effect). As can be seen in the first row of Table 2, in the case of hit-related TEPR peaks, both the direct and the indirect effects are significant. This suggests that one part of the stability in task performance can be captured by hit-related TEPRs, but the mediation is only partial: variability in hit-related TEPR peaks cannot capture all sources that contribute to the correlation between task performance in both sessions. For TEPRs triggered by CRs, only the direct effect is significant (see the last two second row in Table 2). This means that CR-related TEPR peaks do not seem to capture the individual stability in task performance.

**Table 2:** Mediation analyses.

	Mediation model			Direct effect			Indirect effect		
Model	IDV (of Session 1)	DV (of Session 2)	Mediator (composite score)	Effect	S.E	CI_95_	Effect	S.E	CI_95_
1	Sensitivity (*d*_L_)	Sensitivity (*d*_L_)	TEPR for Hits	**0.30**	**0.14**	**0.02–0.58**	**0.11**	**0.29**	**0.07–0.32**
2	Sensitivity (*d*_L_)	Sensitivity (*d*_L_)	TEPR for CRs	**0.37**	**0.14**	**0.08–0.65**	0.04	0.04	− 0.02–0.13
3	TEPR for Hits	TEPR for Hits	Sensitivity (*d*_L_)	**0.38**	**0.15**	**0.07–0.69**	0.12	0.09	− 0.03–0.32
4	TEPR for CRs	TEPR for CRs	Sensitivity (*d*_L_)	**0.65**	**0.11**	**0.43–0.87**	0.03	0.04	− 0.01–0.11

IDV: Independent variable; DV: Dependent variable; S.E.: Standard error; CI: Confidence interval; CRs: Correct rejections; Bold font marks effects with a confidence interval not containing zero

Furthermore, we investigated whether the between-sessions correlations demonstrated in the case of the pupil size metrics are mediated by individual differences in task performance, measured by the mean score of sensitivity (*d*_L_, averaged over the two sessions). As can be seen in the last two rows of Table 2, only the direct pathway is significant, whereas the indirect pathway is not. This means that the correlation between the pupil size metrics is not significantly mediated by cognitive ability.

This pattern of results shows that whereas there is evidence suggesting that TEPRs triggered by hits capture the underlying cognitive ability determining n-back performance (see model 1 in Table 2), the stability in pupil size metrics is definitely moderated by factors that are not related to individual differences in cognitive ability (see models 3–4 in Table 2).

## Interim discussion

In our first study, we could demonstrate the temporal stability of pupil size measures: values of TEPR peaks, baseline pupil size variability and mean showed significant and moderate correlations between the two sessions, which were 2–3 weeks apart. In contrast, we could only partially confirm our predictions regarding the validity of pupil size measures as indices of individual differences in controlled attention: pupil size measures were correlated with task performance in some, but not in all cases. Finally, using mediation analyses, we showed that the temporal stability of pupil size measures is caused by both cognitive and non-cognitive factors. In the following, we will delineate this pattern of results in more detail.

Our first research aim was to replicate previous results showing a link between pupil size measures and task performance in the n-back task^34,36^. Interestingly, the expected correlation with sensitivity (*d*_L_) emerged only in the case of hit-related TEPRs, whereas we could not demonstrate significant correlations for the other pupil size metrics. Note that we found a non-significant tendency for a positive correlation between task performance and TEPRs for CRs in session 2. Further evidence for a link between CR-related TEPR and task performance comes from the finding that, when mean scores were calculated across the two measurements, sensitivity (*d*_L_) was positively correlated with the mean peak TEPR for both hits and CRs—thus the inability to demonstrate a link between CR-related TEPRs and task performance might be caused by low statistical power. To sum up, we were able to partly replicate previous findings^23,35,38,53^ regarding the link between n-back task performance and associated pupil responses for TEPR-related measures, but not for baseline pupil size mean or variability.

Despite our partial failure to replicate previous results concerning the link between pupillary measures and task performance, we could find support in relation to our second research aim: we demonstrated that both the magnitude of task-evoked pupil responses and the variability in baseline pupil size exhibit some degree of temporal consistency. Specifically, good test–retest reliability was evidenced for baseline pupil size mean (ρ = 0.787, *ICC* = 0.813), variability (ρ = 0.579, *ICC* = 0.802) and TEPRs accompanying CRs (ρ = 0.673; *ICC* = 0.778), whereas moderate test–retest reliability was observed for hit-related TEPRs (ρ = 0.413, *ICC* = 0.555). A somewhat lower reliability of this latter measure might be attributable to the rather low statistical power inherent in our design, as there were only 13 hit trials, which might have allowed a higher amount of noise in TEPR peak averging.

Finally, we also conducted a set of mediation analyses to investigate whether the temporal stability in cognitive performance and pupil size metrics, respectively, is caused by the same underlying source. First, we found that individual differences in hit-related TEPRs partially mediate the correlation between task performance at both sessions. This suggests that individual differences in this pupil metric capture stable individual differences in n-back task performance (i.e. they might be sensitive to the underlying cognitive ability). Importantly, the mediation is partial; that is, a part of the variance in cognitive ability underlying n-back performance is independent from the pupil response. Second, we could not find significant mediation in the case of CR-related TEPRs. Thus, we could not demonstrate that this metric is sensitive to stable individual differences in the n-back task. Importantly, this null result does not mean that these pupil size measures are insensitive to individual differences. It might also be the case that due to our underpowered design, we simply could not demonstrate them (i.e. type II error). Third, we also showed a clear and highly significant direct effect for the correlation between the two pupil metrics, when investigating the mediation of individual differences in n-back performance. This pattern suggests that the variance in pupil size metrics, which is responsible for the demonstrated temporal stability, is not necessarily related to the psychophysiological footprint of individual differences in attentional allocation.

To sum up, only a subset of our hypotheses could be justified—as argued in the above sections, this might be the case because low statistical power in our measurement might have been an issue. Note that due to our aim to design a short, potentionally clinically applicable task, our paradigm comprised of only 100 trials with 13 target trials. Accordingly, measurement noise in pupil size metrics might have remained at such a level that a higher number of participants, or a higher number of trials would have been necessary to firmly detect all the predicted effects. To prove this, we ran a second study with the aim of increasing statistical power: instead of one, participants completed four blocks of n-back trials (each block consisted of 100 trials with 13 target trials). Besides this, to improve our design, the following additional changes were made:

First, participants had to press a response button only when they detected an n-back case—in other cases, no response was required. With this modification, we aimed to dissociate pupil dilation associated with the motor response from cognitively driven pupil responses: if TEPR peaks for both hits and CRs show correlation with the behavioral performance, then this correlation cannot be solely attributed to motor responses (as none were required for CRs).

Second, instead of visual, we used auditory stimulus presentation. Although there was only a minimal change in the visual input in experiment 1 when the new stimulus appeared, even this could have introduced visual artefacts on pupillary activity—this might explain the somewhat low magnitude of the evoked pupil responses triggered by CRs, and also the slighlty atypical transient dip on the pupil size curves between 500–1000 ms (see Fig. 4a,b).

Third, we made two additional minor methodological modifications to improve the consistency of our design: Instead of using a variable delay of 2–3 weeks, an identical delay interval of one week was used for every participant. Furthermore, not only pre-trial baseline pupil size was evaluated, but also the mean pupil diameter at the beginning of the experiment, during a 15 s long task-free period, when participants were only instructed to look at the centre of the screen. We will term this measure pre-experimental baseline, and will use this measure alongside the pre-trial baseline mean pupil size. Whereas both methods have been applied previously as a measure of individual differences in baseline pupil size^62,65^, pre-experimental baseline was used in the majority of studies, as it is not influenced by any task-relevant processing.

## Experiment 2—using a long version of the n-back task

### Methods

#### Participants

Data were collected from 45 university students (31 female, *M*_age_ = 21.4 years, *SD*_age_ = 3.9, Range = 18–37). They received monetary compensation or course credits for their efforts. They were restricted to drink caffeinated beverages within six hours or smoke within two hours of the start of the experiment, and all of them reportedly adhered to these constraints. The experiment was carried out in two sessions, exactly seven days apart in all cases. Participants were treated in accordance with the declaration of Helsinki, and the experiment was approved by the United Ethical Review Committee for Research in Psychology, Hungary.

We applied very similar exclusion criteria to the participants of this experiment, as the one we used in experiment 1. One important difference is that this time we used a variant of the task that needed no keypress responses for non-target trials. Accordingly, we could not determine an exclusion criterion based on the number of missed trials (keypress omission). To mitigate this issue, here we excluded participants whose number of false alarms trials laid more than 3 standard deviations away from the sample mean (calculated separately for both sessions). This yielded the exclusion of one participant in both the first and in the second session. We did not exclude any participant due to data quality issues, interpolation percentage values were moderate (*M*_session1_ = 4.05%, *SD*_session1_ = 3.72; *M*_session2_ = 4.91%, *SD*_session2_ = 4.40). However, we had to exclude one participant from the first session because they erroneously performed the third block of the task twice. Based on further exclusion criteria (same as introduced in experiment 1) concerning phasic pupil response peaks and baseline pupil size variability, we excluded one participant from the first session and two from the second session.

After the exclusions, the final sample size was N = 42 for session 1 (28 female, *M*_age_ = 21.1 years, *SD*_age_ = 3.2, Range = 18–36), and N = 42 for session 2 (28 female, *M*_age_ = 21.1 years, *SD*_age_ = 3.2, Range = 18–36; groups not identical). For between-sessions analyses, we excluded participants listwise, meaning that one was excluded if their data were excluded for any of the two sessions. This resulted in a sample size of N = 40 for between-sessions analyses (26 female, *M*_age_ = 21.1 years, *SD*_age_ = 3.2, Range = 18–36).

#### Design and procedure

We used the same laboratory settings and devices as for experiment 1. The design was also similar: participants performed an identical 2-back task with one week delay twice in the laboratory, however, the 2-back paradigm was changed in several aspects. First, in both sessions we used 400 trials, in four blocks of 100 trials, separated with short breaks. We also introduced a 10 trials long practice block at the beginning of the task. Furthermore, we added a 15 second long period at the beginning of the task (after practice), during which participants needed to attend to a fixation cross visible in the center of the screen—this was added to record their pre-experimental baseline pupil sizes at both experimental sessions. Furthermore, whereas we used the same numeric stimuli, this time they were presented in an auditory manner, using earphones. The 3200 ms time between stimulus onsets was unchanged however, and the same fixation cross was visible throughout all trials, eliminating any possible visual response contamination on TEPRs. Also, in experiment 2, we instructed participants to perform keypress responses only when they notice an n-back trials, whereas in all other cases, no response was required—this ensured that motor response planning or execution do not influence pupil responses during non-target trials. Similarly to experiment 1, targets occurred in 13% of all trials, and were presented in the same pseudorandom order for all participants. The same sequence of 400 trials was used in both experimental sessions. Notably, the first two trials of each trial block were not taken into TEPR averaging, phasic peak or pre-trial baseline calculations, as they were not typical n-back trials (see also explanation in experiment 1).

#### Data preparation

Data preparation was carried out in the same way as in case of experiment 1. However, here we not only computed event-related pupillary metrics, but the pre-experimental baseline pupil diameter as well. For this we applied the same data curation methods as what were used for the event-related data extraction proccess (cutting out blinks, and interpolated resampling at 50 Hz, Savitzky-Golay filtering), then we did not break down the recording to epochs, but simply took the average pupil size value over the designated 15 s period while the participant was instructed to look at a static fixation cross screen at the start of the experiment.

#### Statistical analyses

In general, we employed the same statistics and analytical steps as in experiment 1. However, there were minor differences: First, besides computing the pre-trial baseline mean value, we also computed a pre-experimental baseline pupil size measure—this value was measured at the beginning of each session in experiment 2. Second, the time-point analysis of TEPRs suggested that task behavior is strongly correlated with task performance during the trial period preceding the peak (i.e. between 500–1000 ms after stimulus onset). Thus, besides computing mean dilation values for the period of 1000–1500 ms (i.e. peak values), we also computed the mean dilation values for the preceding 500 ms duration (500–1000 ms), for both hits and CRs. We will refer to these new measures as *pre-peak* dilation values. Third, as zero-order correlations with task behavior were more informative with the pre-peak (500–1000 ms), as compared to the peak values (1000–1500 ms), in the results section, mediation analyses with the former variables are reported (mediation analyses with the latter are included in the Supplementary Material). Descriptive statistics for the different pupil size metrics are reported in the Supplementary Material, in Table S2.

### Results

Descriptive statistics of behavioral metrics in experiment 2 are summarized in Table 3. Participants performed well, and even a few of them reached the ceiling in the second session, but the distribution was still appropriate for later correlative analyses.

**Table 3:** Descriptive statistics.

	Session 1						Session 2					
Metric	Hit rate	FA rate	Sensitivity (*d*_L_)	Bias (*c*_L_)	Hits RT (ms)	CRs RT (ms)	Hit rate	FA rate	Sensitivity (*d*_L_)	Bias (*c*_L_)	Hits RT (ms)	CRs RT (ms)
N	42						42					
M	0.895	0.013	7.550	1.217	1029	–	0.899	0.007	8.552	1.341	1094	–
SD	0.084	0.013	2.141	0.707	156	–	0.104	0.009	2.544	0.714	198	–
Min	0.654	0.001	3.463	− 0.540	730	–	0.519	0.001	3.803	− 1.093	667	–
Max	0.999	0.056	12.733	2.601	1480	–	0.999	0.044	13.814	2.435	1673	–

Reaction times are in milliseconds. Individual hit rates were calculated by dividing the number of hits by the total number of possible targets (52), limited at 0.001 from below and at 0.999 from above. Individual false alarm rates were calculated by dividing the number of hits by the total number of possible non-targets (340, excluding the first two trials of each block, as they needed no response), limited at 0.001 from below and at 0.999 from above. FA: False alarm; CRs: Correct rejections; RT: Response time.

Surprisingly, we found a small but significant slowing in reaction times to target hits in the second session as compared to the first one (t(39) = -3.389, *p* = 0.002, d = -0.536), but also a significant improvement in sensitivity (*d*_L_) scores (t(39) = -3.514, *p* = 0.001, d = -0.556).

#### The link between pupillary measures and task performance—the question of validity

##### Session 1

**Fig. 6:**
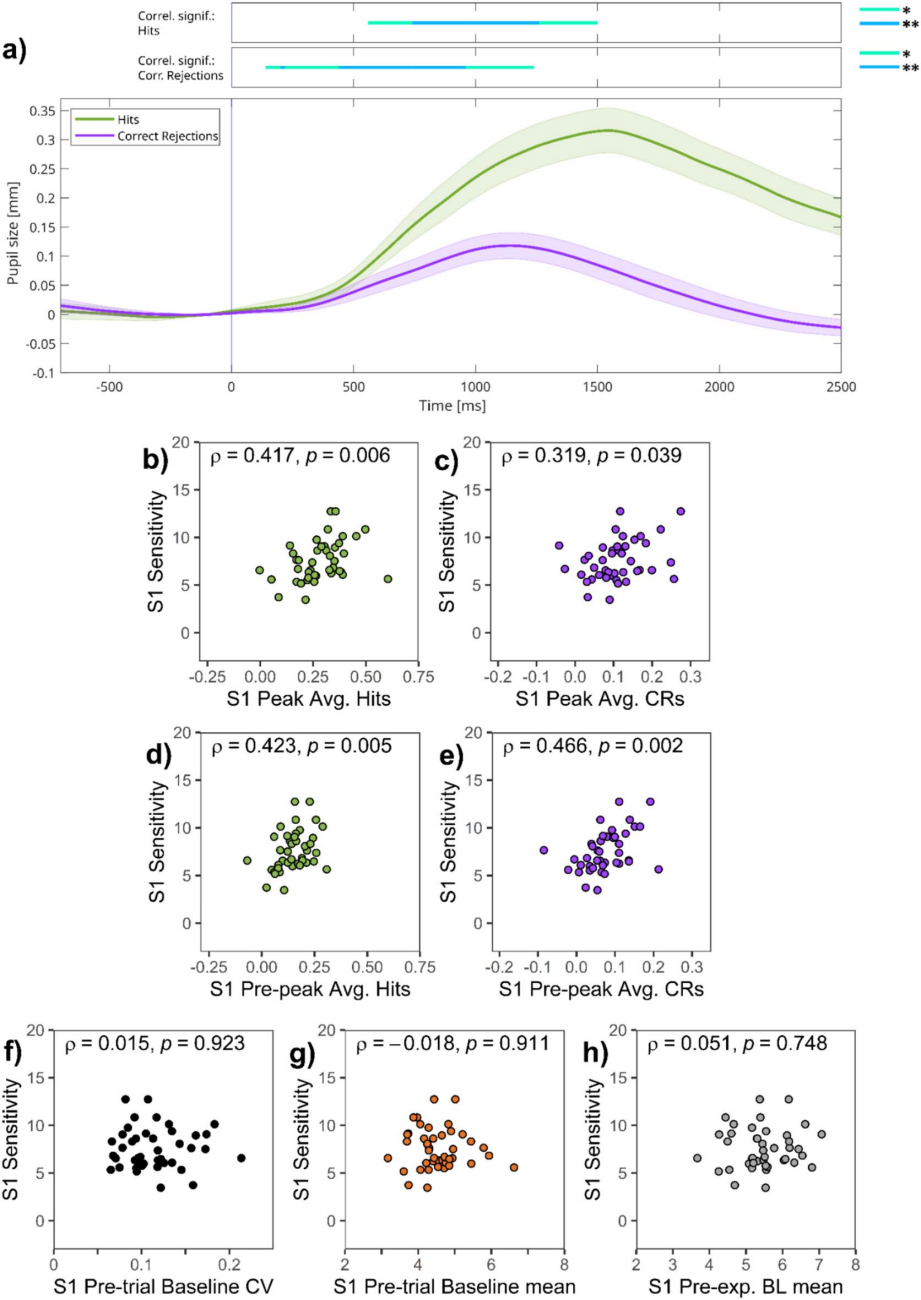
Session 1 of experiment 2—(**a**) Task-evoked pupillary responses and (**b**–**h**) correlations of pupillary responses and task performance. *Note:* (**a**) Grand average task-evoked pupillary response (TEPR) curves for target hits and correct rejection (CR) trials. Pupil size curves are stimulus-aligned, baseline-corrected for the preceding 200 ms, and averaged across all trials and participants. The bars on the top represent regions of significance, regarding time-course analyses, correlating the event-related pupil response curves and sensitivity (*d*_L_) scores in session 1 (*N* = 42). Pupil size metrics are in millimeters. Colored areas represent 95% confidence intervals. (**b**–**h**): Scatter plots showing the link between sensitivity (*d*_L_) and: (**b**) TEPR peak (1000–1500 ms average after stimulus onset) values for target hits, (**c**) TEPR peak (1000–1500 ms average after stimulus onset) values for CRs, (**d**) TEPR pre-peak (500–1000 ms average after stimulus onset) pupil size for target hits, (**e**) TEPR pre-peak (500–1000 ms average after stimulus onset) pupil size for CRs, (**f**) pre-trial baseline pupil size variability (CV: Coefficient of variation), (**g**) pre-trial baseline pupil size mean, and (**h**) pre-experimental baseline (BL) pupil size mean.

Pupil responses during hit and CR trials in session 1 are presented in Fig. 6a. Hits evoked a similar response to the one observed in experiment 1 (green line on Fig. 6a), and pupil responses for CRs were again smaller than hit-related dilations (purple line on Fig. 6a).

We inspected the correlation between sensitivity (*d*_L_) and TEPR peak average of the 1000–1500 ms interval after stimulus onset. Sensitivity (*d*_L_) correlated significantly with TEPR peak values in case of target hits (ρ = 0.417, *p* = 0.006; Fig. 6b) and CRs (ρ = 0.319,* p* = 0.039; Fig. 6c) as well. We also investigated how sensitivity (*d*_L_) correlates with the amount of pupil dilation at each time point after stimulus onset. Time-course analysis was conducted (as shown on top bars of Fig. 6a, see Methods of experiment 1 for details; see also Figure S4 in the Supplementary Material). Several periods of the trial duration correlated significantly between sensitivity (*d*_L_) and momentary pupil size change. This is congruent with the results of our analysis concerning TEPR peak values above.

Interestingly, the results of the time-course analysis suggest that in the case of CRs, the correlation between task-performance and pupil dilation is stronger in the period preceding the peak pupil response. Thus, as a post-hoc analysis, we also computed mean values for this pre-peak period. Confirming this observation, the correlations with task performance were stronger in the case of CRs (ρ = 0.466, *p* = 0.002; Fig. 6e), whereas it did not change for hits (ρ = 0.423, *p* = 0.005; Fig. 6d).

We also investigated the correlation between mean pre-trial baseline pupil size, its variability, as well as pre-experimental pupil size with task performance. Contrary to our expectations, we found no correlation with n-back sensitivity (*d*_L_) in case of either the variability (ρ = 0.015, *p* = 0.923; Fig. 6f) or the mean (ρ = -0.018, *p* = 0.911; Fig. 6g) of pre-trial baseline pupil size. Pre-experimental baseline pupil diameter did not predict sensitivity (*d*_L_) either (ρ = 0.051, *p* = 0.748; Fig. 6h). Interestingly, pre-trial and pre-experimental baseline pupil size were strongly correlated (ρ = 0.766, *p* < 0.001; see also Supplementary Material, Figure S7a).

To sum up, we could demonstrate significant correlation between task performance and TEPRs for both hits and CRs, whereas metrics of baseline pupil size were not related to performance.

##### Session 2

**Fig. 7:**
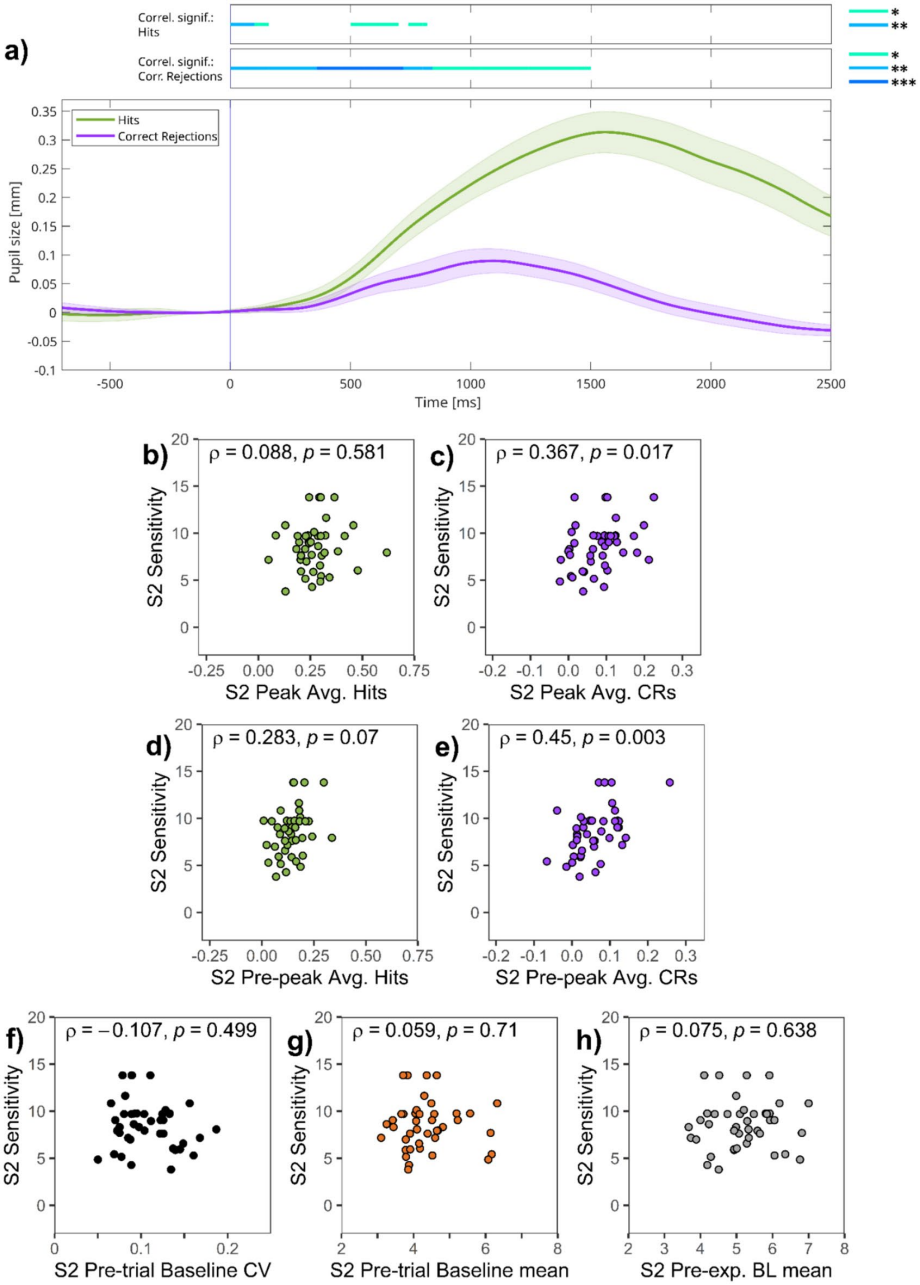
Session 2 of experiment 2—(**a**) Task-evoked pupillary responses and (**b**–**h**) correlations of pupillary responses and task performance. *Note:* (**a**) Grand average task-evoked pupillary response (TEPR) curves for target hits and correct rejection (CR) trials. Pupil size curves are stimulus-aligned, baseline-corrected for the preceding 200 ms, and averaged across all trials and participants. The bars on the top represent regions of significance, regarding time-course analyses, correlating the event-related pupil response curves and sensitivity (*d*_L_) scores in session 1 (*N* = 42). Pupil size metrics are in millimeters. Colored areas represent 95% confidence intervals. (**b**–**e**): Scatter plots showing the link between sensitivity and: (**b**) TEPR peak (1000–1500 ms average after stimulus onset) values for target hits, (**c**) TEPR peak (1000–1500 ms average after stimulus onset) values for CRs, (**d**) TEPR pre-peak (500–1000 ms average after stimulus onset) pupil size for target hits, (**e**) TEPR pre-peak (500–1000 ms average after stimulus onset) pupil size for CRs, (**f**) pre-trial baseline pupil size variability (**g**) pre-trial baseline pupil size mean, and (**h**) pre-experimental baseline (BL) pupil size mean.

TEPRs show similar characteristics (Fig. 7a) to those osberved in session 1. We first inspected the correlation between sensitivity (*d*_L_) and TEPR peak average during 1000–1500 ms after stimulus onset. Peak pupil responses for hits did not correlate with sensitivity (ρ = 0.088, *p* = 0.581; Fig. 7b), but peak pupil dilation for CRs did (ρ = 0.367, *p* = 0.017; Fig. 7c). These correlations were also computed on a moment-to-moment basis, using time-course analyses (bars on top of Fig. 7a; see also Figure S5 in the Supplementary Material). Interestingly, similar to session 1, this analysis again suggested that the magnitude of pupil dilation correlates stronger with task performance during the pre-peak period between 500–1000 ms. Due to this, we again computed mean pupil dilation values for this pre-peak period. Correlations of pupil dilation observed during this pre-peak period (500–1000 ms) were noticeably higher in the case of both target hit (ρ = 0.283, *p* = 0.070; Fig. 7d) and CR trials (ρ = 0.450, *p* = 0.003; Fig. 7e).

The correlation between mean pre-trial baseline pupil size, its variability, and pre-experimental pupil size with task performance was also investigated. Contrary to our expectations, we found no correlation between sensitivity (*d*_L_) and either the variability (ρ = -0.107, *p* = 0.499; Fig. 7f) or the mean (ρ = 0.059, *p* = 0.710; Fig. 7g) of pre-trial baseline pupil size. Pre-experimental baseline pupil size did not correlate with sensitivity (*d*_L_) either (ρ = 0.075, *p* = 0.638; Fig. 7h). Again, we found a significant correlation between pre-trial and pre-experimental baseline pupil size (ρ = 0.826, *p* < 0.001; see Supplementary Material, S7b).

To sum up, similarly to experiment 1, we found that only pupil size metrics associated with TEPRs triggered by hits and CRs were related to task performance. Furthermore, this relation was linked to the period between 500–1000 ms after the presentation of the new stimulus.

#### Correlation of pupillary responses between two sessions—the question of test–retest reliability

**Fig. 8:**
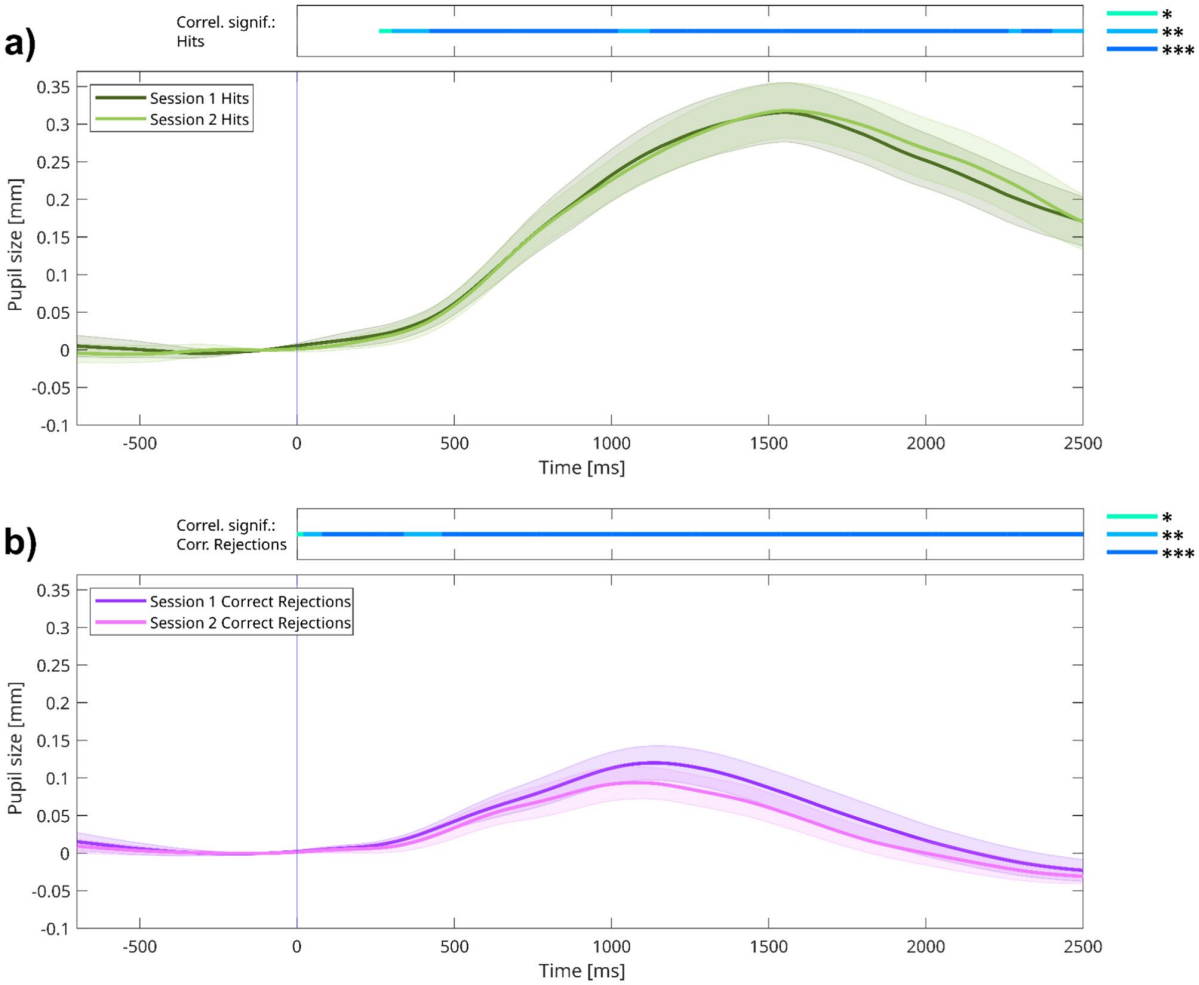
Task-evoked pupil responses of sessions 1 and 2 in experiment 2, for (**a**) hits and (**b**) correct rejections (CRs). *Note:* (**a**–**b**): Grand average task-evoked pupillary response (TEPR) curves for target hit and correct rejection (CR) trials, depicted for both sessions. Pupil size curves are stimulus-aligned, baseline-corrected for the preceding 200 ms, and averaged across all trials and participants. The bars on the top represent regions of significance, regarding time-course analyses, correlating the pupil size curves at the two sessions (*N* = 40). Colored areas represent 95% confidence intervals.

We visualized TEPR for hits and CRs for both sessions on Fig. 8a,b. Pupillary responses exhibit high similarity between session 1 and session 2, with respect to peak magnitude and latency as well. Importantly, when comparing responses from both sessions between experiment 1 and experiment 2, it is noticeable that the transient decrease in pupil dilation values observed in experiment 1 between 500–1000 ms is not present here (compare Figs. 4a,b and 8a,b). This suggests that this temporary dip in pupil responses observed in experiment 1 might have been caused by the visual stimulus presentation, and thus disappeared when we changed stimulus presentation to auditory in experiment 2.

**Fig. 9:**
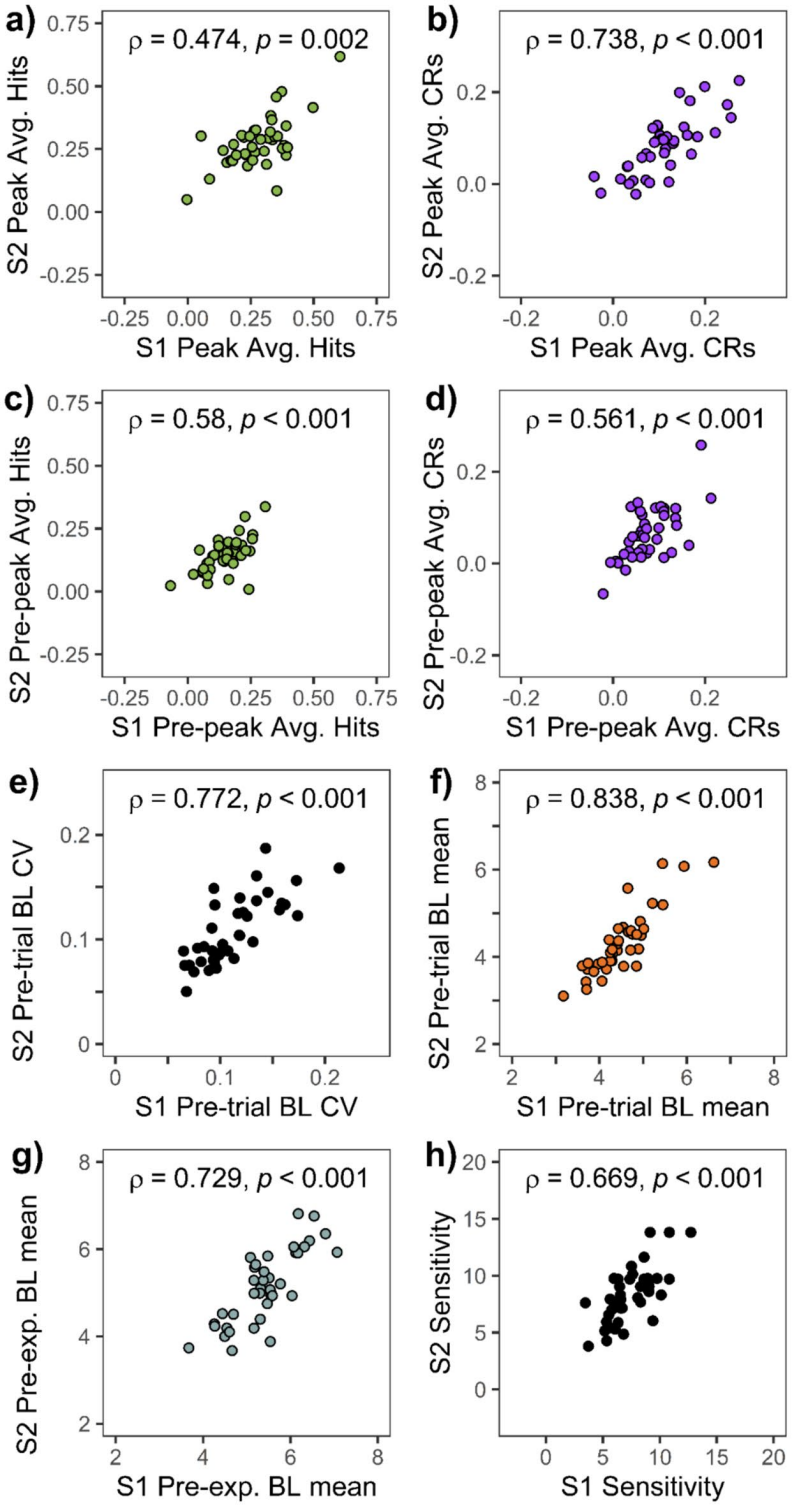
Between-sessions correlations of (**a**–**g**) pupil responses and (**h**) task performance. *Note:* Scatter plots showing the correlation between the values measured at the two sessions of experiment 2: TEPR peaks (1000–1500 ms average after stimulus onset) during (**a**) target hits and (**b**) correct rejections (CRs). Pre-peak (500–1000 ms average after stimulus onset) pupil size during (**c**) target hits and (**d**) correct rejections. Pre-trial baseline (BL) pupil size (**e**) variability (CV: Coefficient of variance) and (**f**) mean. (**g**) Pre-experimental baseline pupil size mean. (**h**) Sensitivity (*d*_L_) values. Pupil size metrics are in millimeters. (*N* = 40).

First, we investigated the correlation of TEPR peaks from the two sessions separately for the two trial types. The analysis showed that TEPR peak values for target hits from session 1 and session 2 were strongly correlated (ρ = 0.474,* p* = 0.002; *ICC* = 0.785, 95% CI: 0.593–0.887; Fig. 9a). An even higher degree of between-sessions correlation was found for TEPR peak values during CR trials (ρ = 0.738, *p* < 0.001; *ICC* = 0.830, 95% CI: 0.576–0.922; Fig. 9b) across participants. The test–retest correlations are comparable, when not the peak (1000–1500 ms) but the pre-peak period is examined (500–1000 ms): mean pupil dilation values show good test–retest reliability both in case of hits (ρ = 0.580, *p* < 0.001; *ICC* = 0.771, 95% CI: 0.565–0.879; Fig. 9c) and CRs (ρ = 0.561, *p* < 0.001; *ICC* = 0.778, 95% CI: 0.584–0.882; Fig. 9d).

Using time-course analyses, we also investigated the between-sessions correlations of individual TEPR curves in a moment-to-moment manner (bars on top of Fig. 8a,b; see also Figure S6 in the Supplementary Material). These analyses underlined the results of the TEPR peak analysis, revealing significant and strong correlations for hits and CRs during the stimulus presentation interval.

Both the variability (ρ = 0.772, *p* < 0.001;*ICC* = 0.859, 95% CI: 0.734–0.925; Fig. 8e), and the mean (ρ = 0.838, *p* < 0.001; *ICC* = 0.915, 95% CI 0.801–0.959; Fig. 8f) of pre-trial baseline pupil size values showed excellent test–retest reliability, and this was also the case for the pre-experimental baseline mean pupil size (ρ = 0.729, *p* < 0.001; *ICC* = 0.855, 95% CI: 0.654–0.932; Fig. 8g). As expected, sensitivity (*d*_L_) correlated between sessions (ρ = 0.669, *p* < 0.001; *ICC* = 0.794, 95% CI 0.535–0.901; Fig. 8h).

To sum up, we found that all five pupil size measures showed good test–retest reliability over a period of one week, and in the case of the TEPRs, the time-course analysis suggests that this correlation is present throughout the trial duration.

#### Mediation analyses

As the next step, we conducted a mediation analysis to examine whether the mean scores of the pupil size metrics (averaged across the two sessions) mediate the relationship between task performance in the two sessions (see Fig. 1). This analysis allowed us to estimate the extent to which the association between the independent and dependent variables is accounted for by a third, mediating variable (indirect effect), and the extent to which it remains independent of the mediator (direct effect). Similarly to experiment 1, only those variables were entered in this analysis, which demonstrated a meaningful zero-order correlation with task performance: thus, mediation analysis was conducted using pupil dilation values computed for pre-peak (500–1000 ms after stimulus onset) and peak (1000–1500 ms after stimulus onset) periods, for both hits and CRs.

As a first step, we again computed mean values from the individual metrics and scores from both sessions. Similarly to experiment 1, mean value of the sensitivity score was correlated with the mean values of the pupil size metrics (pre-peak during hits: ρ = 0.432, *p* = 0.005; pre-peak during CRs: ρ = 0.494, *p* < 0.001; peak during hits: ρ = 0.271, *p* = 0.091; peak during CRs: ρ = 0.347, *p* = 0.028).

In the case of the peak period, none of the mediation effects were significant, thus we could not find any evidence for the claim that there would be a shared variance responsible for the temporal stability of task performance and pupil dilation (see results in Table S3 in the Supplementary Material). In contrast, the results of the mediation analysis conducted using the measures of the pre-peak period showed significant mediation effects: in the case of CRs, the correlation between the pupil size measures was partially mediated by the mean of the sensitivity (*d*_L_) scores from the two sections (see Table 4, second row). And vice versa: the correlation between the sensitivity (*d*_L_) scores from the two sessions were partially mediated by the mean of pre-peak value of CR-related pupil dilation from the two sessions (see Table 4, fourth row). This pattern of results suggests that there might be a common factor contributing to the temporal stability of both task performance and pupil dilation—one might suspect that this shared common variance can be attributed to underlying neural processes, which influence both task performance and pupil responses. Note also, that similar to experiment 1, the mediation is only partial. That is, the temporal stability is determined also by different factors (e.g. measurement related factors in the case of pupil size metrics).

**Table 4:** Mediation analyses.

	Mediation model			Direct effect			Indirect effect		
Model	IDV (of Session 1)	DV (of Session 2)	Mediator (composite score)	Effect	S.E	CI_95_	Effect	S.E	CI_95_
1	Sensitivity (*d*_L_)	Sensitivity (*d*_L_)	TEPR for Hits	**0.80**	**0.14**	**0.52–1.08**	0.07	0.05	− 0.01–0.17
2	Sensitivity (*d*_L_)	Sensitivity (*d*_L_)	TEPR for CRs	**0.72**	**0.14**	**0.44–1.01**	**0.14**	**0.07**	**0.03–0.31**
3	TEPR for Hits	TEPR for Hits	Sensitivity (*d*_L_)	**0.53**	**0.13**	**0.27–0.78**	0.04	0.06	− 0.05–0.21
4	TEPR for CRs	TEPR for CRs	Sensitivity (*d*_L_)	**0.59**	**0.15**	**0.29–0.89**	**0.14**	**0.08**	**0.02–0.35**

IDV: Independent variable; DV: Dependent variable; S.E.: Standard error; CI: Confidence interval; CRs: Correct rejections; Bold font marks effects with a confidence interval not containing zero.

## Discussion

In two experiments, we investigated the validity and reliability of pupil size metrics measured during an n-back task, which relies heavily on the ability to update working memory contents. In the first experiment, we used a rather short version of the task consisting of 100 trials (duration about five minutes), and we found only inconclusive evidence regarding the validity and reliability of pupil size measures. Thus, in a new experiment, we used a task consisting of 400 trials (duration about 20 min). Using this longer task version, we found more convincing evidence for the validity and reliability of pupil size metrics, as a measure of individual differences in neural processing underlying working memory updating. Importantly, in line with previous research^34,36^, we showed that the magnitude of TEPRs during the n-back task are correlated with behavioral performance, and we also demonstrated temporal stability of these TEPRs by demonstrating significant test–retest correlations. In our mediation analyses, we found evidence—at least for one specific measure—for the claim that stable individual differences in pupil size measures can be linked to stable individual differences in n-back performance. Importantly, however, our mediation analyses also showed that a large part of the temporally stable part of pupil responses is independent of behavioral performance, and is most likely related to non-cognitive factors. In the case of tonic pupil size measures (baseline pupil size mean and variability), we found excellent reliability, but poor validity (i.e. low correlation with task performance). This latter might be explained by specifics of our task design. Finally, we varied several aspects of the two experiments (trial length, visual vs. auditory presentation, measurement of pupil size indices), and these changes considerably affected the validity and reliability of different pupil size measures. In the following, we provide a detailed summary on the pattern of results from the two experiments, with an interpretation of what they might suggest regarding the use of pupillometry as an individual difference measure.

### Summary of the results

#### The question of validity

In experiment 1, n-back performance was related to TEPRs associated with hits (session 1: ρ = 0.266,* p* = 0.033; session 2: ρ = 0.403, *p* = 0.001), and was unrelated to the variability (session 1: ρ = 0.083, *p* = 0.514; session 2: ρ = 0.056, *p* = 0.661) and mean (session 1: ρ = -0.130, *p* = 0.305; session 2: ρ = -0.014, *p* = 0.915) of pre-trial baseline pupil size. The pattern of correlation was inconclusive with respect to TEPRs associated with CRs (session 1: ρ = 0.099,* p* = 0.436; session 2: ρ = 0.244, *p* = 0.054).

In experiment 2, due to an increase in statistical power (i.e. more trials), we could find a more conclusive pattern of results regarding TEPRs. Task performance was significantly related to peak dilation values (computed for the period 1000–1500 ms after stimulus onset) in the case of CRs (session 1: ρ = 0.319,* p* = 0.039; session 2: ρ = 0.367, *p* = 0.017), and it was also related to peak pupil responses during hits in the first, but not in the second session (session 1: ρ = 0.417, *p* = 0.006; session 2: ρ = 0.088, *p* = 0.581). Interestingly, if mean dilation values were computed for the pre-peak period between 500–1000 ms, then the correlation with sensitivity (*d*_L_) stayed significant and strong in session 1, and improved to a non-significant trend with in session 2 (session 1: ρ = 0.423, *p* = 0.005; session 2: ρ = 0.283, *p* = 0.070). Note that the correlations with TEPRs during CRs were also stronger when evaluted during this pre-peak period (session 1: ρ = 0.466, *p* = 0.002; session 2: ρ = 0.450, *p* = 0.003). This pattern suggests that pupillary activity in this time window better reflects task performance. In contrast to phasic measures, tonic pupil size metrics showed poor validity: task performance was unrelated to both the mean (session 1: ρ = -0.018, *p* = 0.911; session 2: ρ = 0.059, *p* = 0.710) and variability (session 1: ρ = 0.035, *p* = 0.826; session 2: ρ = -0.107, *p* = 0.499) of pre-trial baseline pupil size. Furthermore, task-performance was not predicted by pre-experimental baseline pupil size (session 1: ρ = 0.051, *p* = 0.748; session 2: ρ = 0.075, *p* = 0.638).

Thus, to sum up, whereas the results are somewhat mixed, the pattern of our results confirms previous results^34,36^ suggesting that TEPRS show a positive correlation with n-back task performance. Interestingly, in experiment 1, TEPRs related to hits, whereas in experiment 2, TEPRs related to CRs showed stronger correlation with behavioral performance. The results show less evidence for a correlation between task performance and tonic pupil size metrics: sensitivity (*d*_L_) score was not correlated to either the mean or the variability of pre-trial pupil size, and no link was evident regarding pre-experimental pupil baseline pupil size either. We will elaborate on the possible causes of this pattern of results later, in Section "Implications of our findings regarding the use of pupillometry as an individual difference measure".

#### The question of test–retest reliability

In general, we found significant test–retest correlations and evidence for good test–retest reliability between pupil size metrics measured at the two sessions. The magnitude of correlations was somewhat below expectations in experiment 1, but increasing statistical power in experiment 2 resulted in more appropriate reliability estimates. Specifically, correlations were highest for baseline pupil size measures, either computed as the mean of pre-trial pupil sizes (experiment 1: ρ = 0.787, *ICC* = 0.813; experiment 2: ρ = 0.838, *ICC* = 0.915), or measured at the beginning of the experiment (experiment 2: ρ = 0.729, *ICC* = 0.855). Reliability was also good for pre-trial baseline pupil size variability (experiment 1: ρ = 0.579, *ICC* = 0.802; experiment 2: ρ = 0.772, *ICC* = 0.859), whereas it was was somewhat lower for task-evoked responses triggered by hits (experiment 1: ρ = 0.413, *ICC* = 0.555; experiment 2: ρ = 0.474, *ICC* = 0.785) and by CRs (experiment 1: ρ = 0.673, *ICC* = 0.778; experiment 2: ρ = 0.738, *ICC* = 0.830).

#### Mediation analyses

In both experiments, we could show a partial mediation between one specific measure of TEPRs and n-back sensitivity (*d*_L_) score. Specifically, in both experiments, we could demonstrate that the correlation between task performance in the two sessions can be partly explained by a TEPR-related pupil size metric (related to hits in experiment 1 and CRs in experiment 2). We also found evidence for the opposite in experiment 2: the between-session correlation between the pre-peak mean dilation was partly explained by task performance. This suggests that temporally stable individual differences in pupil size measures can be related to individually stable differences in n-back performance. One plausible interpretation for this result is that the variance shared between these measures reflects underlying neural mechanisms that affect both task performance and pupillary responses as well.

Importantly, however, the path measuring direct effects was significant in all our analyses—this shows that a considerable part of the variance in both task performance and pupillary responses is determined by independent factors. Thus, ideally, when designing a task aimed at assessing individual differences in pupillary responses that are informative about cognitive processes, one should strive for a task in which pupillary measures show a significant and large mediation effect on the between-session correlations of task performance measures.

### Implications of our findings regarding the use of pupillometry as an individual difference measure

In general, the pattern of results suggests that task-evoked phasic pupillary dilations might be considered promising targets for assessing individual differences in functioning of neural networks underlying working memory updating. They tend to correlate with behavioral performance, show temporal stability, and can explain temporally stable aspects of behavioral performance. Although, our results show that design and analysis choices play a decisive role in determining whether these indicators can truly be regarded as reliable measures of the aforementioned individual differences.

This is nicely demonstrated by the results regarding the validity of TEPRs. As summarized above, in the two experiments, task performance correlated differently with mean pupil dilation values of the peak period (1000–1500 after stimulus onset): in experiment 1, values for hits, whereas in experiment 2, values for CRs are linked with n-back performance. This difference might be caused by the fact that in experiment 2, the instruction for selective keypresses to n-back targets might have increased the salience of hits and this might have changed the determinants of pupil dilation accompanying hits. One might speculate that the cause for the lower correlations between hit-related TEPRs and behavioral performance in experiment 2 might be that in this case changes in pupil size primarily reflected an orienting response rather than the underlying cognitive control processes.

Additionally, in experiment 2, correlations with task performance were stronger for mean dilation values computed during the pre-peak period (500–1000 ms) than during the peak period (1000–1500 ms). This difference is noteworthy in light of the fact that a possible visual artifact may have affected our pupil size measurements in experiment 1—as can be seen in Fig. 4a,b, a transient decrease in pupil size can be observed between 500–1000 ms. It is possible that the absence of correlations with task performance in this period in experiment 1 was caused by the confounding effects of visual presentation during this time period. Importantly, in both cases, subtle design choices (visual vs. auditory presentation or task instructions) altered the characteristics of the evoked pupil responses, which might have influenced their correlations with task performance. Finally, as no keypress was required for CRs in experiment 2, the demonstrated test–retest reliability and validity of TEPRs associated with CRs cannot be explained by a confounding effect of motor response initiation.

Design choices might have also affected the results related to tonic pupil size measures. Whereas all three measures showed excellent test–retest reliability, they did not predict task performance in either of our experiments. In the case of pre-trial baseline pupil size variability, this might be related to the relative short baseline period of 200 ms. This was chosen with the consideration that we aimed to create a short task version. Note that it is significantly shorter than the baseline period of 2500 ms used by Robison and Garner^36^, who reported a significant negative correlation between pre-trial baseline pupil size variability and n-back performance. The insufficient duration of the baseline period is even more critical in the case of the n-back, as active maintenance is required throughout the task—therefore, controlled attention or mental effort is needed even during the fixation period. Thus, controlled, effortful information processing occurs during the baseline period, which may have additionally limited the adequacy of this measure for assessing baseline, tonic activity.

In the case of pre-trial or pre-experimental baseline pupil size, the lack of significant correlations with task performance might be also explained by methodological details. According to a recent suggestion^63^, controversial results regarding the link between baseline pupil size and cognitive performance may be due to a methodological confound: when pupils are constricted, their size shows less variability, and thus the link between pupil size and cognitive ability can be demonstrated only when the pupils are relatively dilated (but see^64^ for a study, in which no link could be demonstrated with relatively high mean pupil size either). Mean baseline pupil size values in our study were somewhat lower than in previous studies finding a positive correlation^36,62^ (see Supplementary Material, Table S1 and S2), and this might have led to our inability to find a significant correlation. Note also, that the aforementioned studies^62,63^ used tasks assessing complex working memory or fluid intelligence. Whereas these tasks are conceptually similar to the n-back task, they are nevertheless different—and this difference might have also contributed to the lack of effect in our study.

Thus, future research using task designs better optimalized to the measurement of tonic pupil size measures are warranted to test whether our null results are due to these methodological factors. Nevertheless, in the case of these tonic measures, our research demonstrated a clear dissociation of validity and reliability: whereas these metrics showed the highest test–retest correlations, they were not correlated with behavioral performance. Importantly, this dissociation does not mean that pupil size measures cannot be used to detect individual differences in attentional allocation or in the functioning of related brain networks. In our current study we could demonstrate that TEPRs triggered by hits (experiment 1) and CRs (experiment 2) can grasp temporally stable individual differences in attentional allocation (see above). But the mediation analyses also pointed out that the temporal stability of pupil measures also originates from other sources (i.e. physiological factors or participant-specific aspects of measurement noise). Thus, to show that pupil size measures can be used to track temporally stable individual differences in information processing, one has to consider the different sources of variance, which could contribute independently to this temporal stability. Whereas our mediation analysis might show some indication regarding this issue, studies using large sample sizes and more advanced statistical apparatus (e.g. structural equation modelling) are definitely required to investigate how variance related to pupil size measures and cognitive performance covaries both between participants and between sessions.

Finally, our results from the two experiments clearly show that statistical power and sufficient trial number is of utmost importance for pupillometric studies. Due to the rather low signal-to-noise ratio of the pupil signal, researchers interested in individual differences in pupil size should always ensure that their experimental design has sufficient statistical power to yield measures with adequate validity and reliability. In the case of the n-back task, our results suggest that a short, five-minute test format does not provide adequate measurement quality, indicating that a longer task duration is required. Although, for other tasks fewer trials may still prove sufficient. Given that even minor design modifications in our two experiments (e.g., visual vs. auditory presentation) influenced the pattern of results, we conclude that the validity and reliability of pupil size metrics are quite sensitive to the design used—that is, shorter or longer task durations may be adequate in different task contexts.

## Conclusion

Our results suggest that pupil size measures can be valid and reliable indices of individual differences in controlled attention, possibly reflecting neural processes, but short, easy-to-administer versions of cognitive tasks might be underpowered to exhibit appropriate levels of validity and reliability. Furthermore, we also showed that the test–retest reliability of pupil indices might also reflect non-cognitive, measurement-related factors. Thus, when assessing individual differences in pupil size metrics in applied settings, one should take extra care of the methodological challenges presented in the current paper: ideally, both the validity and test–retest reliability of the design should be investigated, short-versions of cognitive tasks should be extensively tested for statistical power, and mediation analyses should ensure that a test–retest correlation between pupil size measures reflects individual differences in cognitive ability and not measurement-specific factors.

## Supplementary Information

Supplementary Information.

## Data Availability

The code for the pupil data preprocessing and the code and data for statistical analyses are publicly available on this OSF link: https://osf.io/gj7xa/
