# Supplementary material for: Investigating the test–retest reliability and behavioral correlates of pupil responses in the n-back task
Source: Sci Rep. 2026 Apr 11;16:17032. doi: 10.1038/s41598-026-46731-3 (PMC13230787; doi:10.1038/s41598-026-46731-3)
Supplement: Supplementary file 1 — Supplementary Information. [file 41598_2026_46731_MOESM1_ESM.docx]

**Supplementary Material**

**Manuscript title:**

**Investigating the test-retest reliability and behavioral correlates of pupil responses in the n-back task**

Gábor László Bényei ^a,b^ (benyeig@edu.bme.hu);
Péter Pajkossy ^a,c^ (pajkossy.peter@ttk.bme.hu)

^a^ Department of Cognitive Science, Faculty of Natural Sciences, Budapest University of Technology and Economics, Műegyetem rkp. 3., H-1111 Budapest, Hungary

^b^ Neurocognitive Research Centre, Nyírő Gyula National Institute of Psychiatry and Addictology, Lehel u. 59., H-1135 Budapest, Hungary

^c^ Cognitive Medicine Research Group, Competence Centre for Neurocybernetics of the Life Sciences Cluster of the Centre of Excellence for Interdisciplinary Research, Development and Innovation of the University of Szeged, University of Szeged

**Supplementary Figure S1:**

Time-course analysis of individual TEPR curves regarding (a) target hit trials and (b) correct rejection trials in experiment 1.


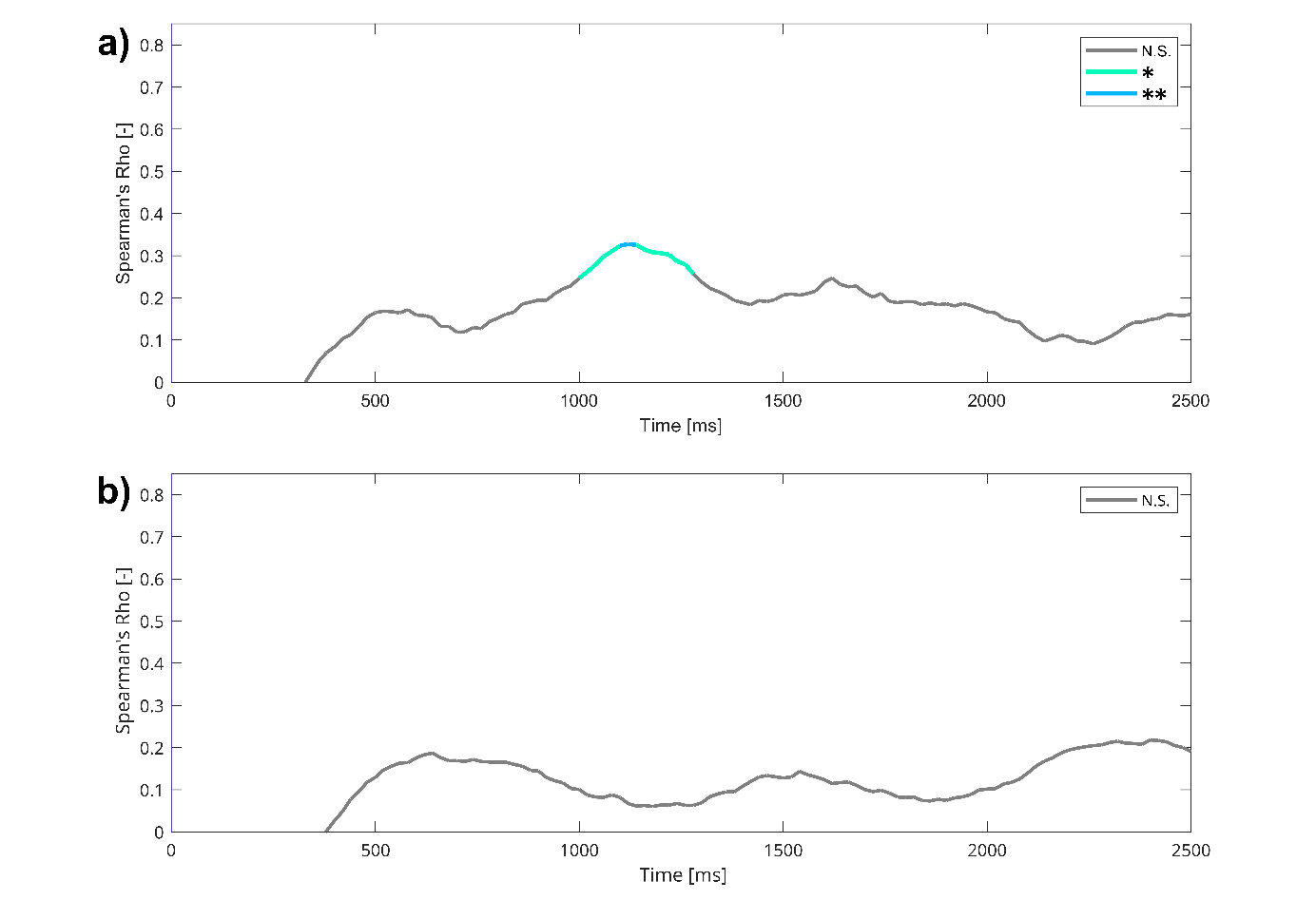


*Note:* For time-course analyses, each baseline-corrected timepoint of a specific TEPR curve in session 1 was correlated with sensitivity (*d*_L_) scores, across all participants (*N* = 64).

*: p < .05

**Supplementary Figure S2:**

Time-course analysis of individual TEPR curves regarding (a) target hit trials and (b) correct rejection trials in experiment 1.


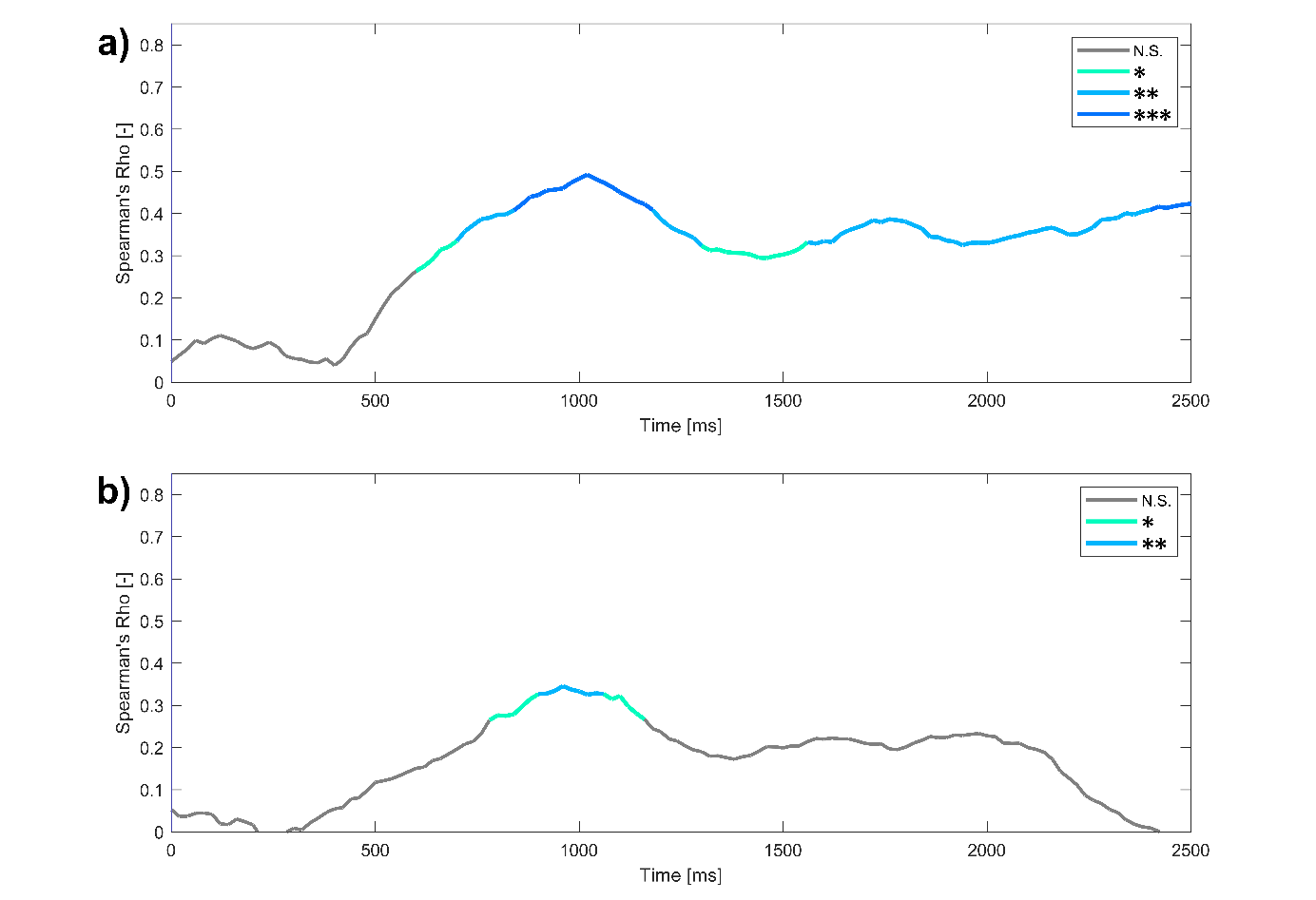


*Note:* For time-course analyses, each baseline-corrected timepoint of a specific TEPR curve in session 2 was correlated with sensitivity (*d*_L_) scores, across all participants (*N* = 63).

*: p < .05; **: p <.01; ***: p < .001

**Supplementary Figure S3:**

Time-course analysis of individual TEPR curves regarding (a) target hit trials and (b) correct rejection trials in experiment 1.


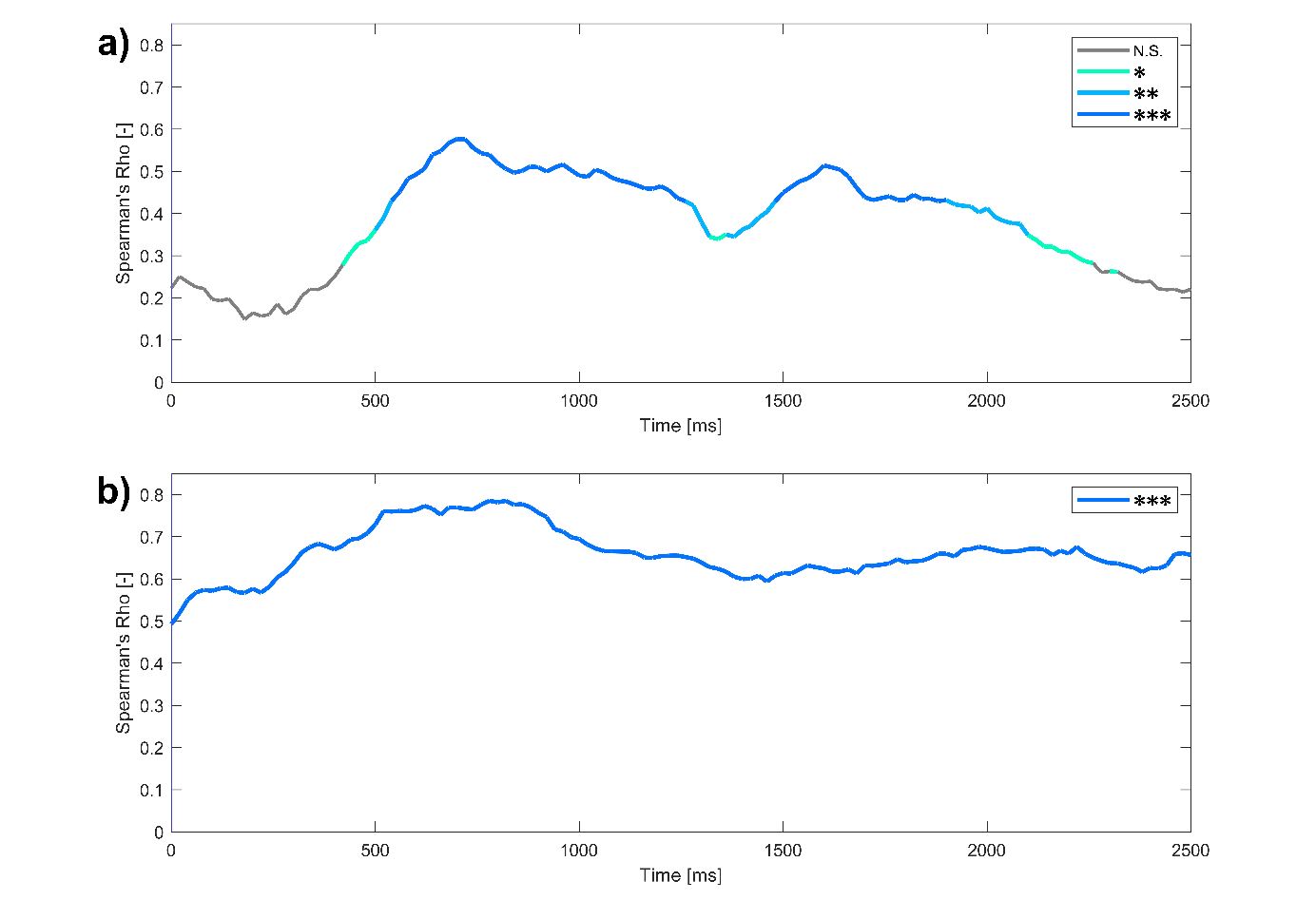


*Note:* For time-course analyses, each baseline-corrected timepoint of a specific TEPR curve in session 1 was correlated with its corresponding timepoint on session 2, across all participants (*N* = 56).

*: p < .05; **: p <.01; ***: p < .001

**Supplementary Figure S4:**

Time-course analysis of individual TEPR curves regarding (a) target hit trials and (b) correct rejection trials in experiment 2.


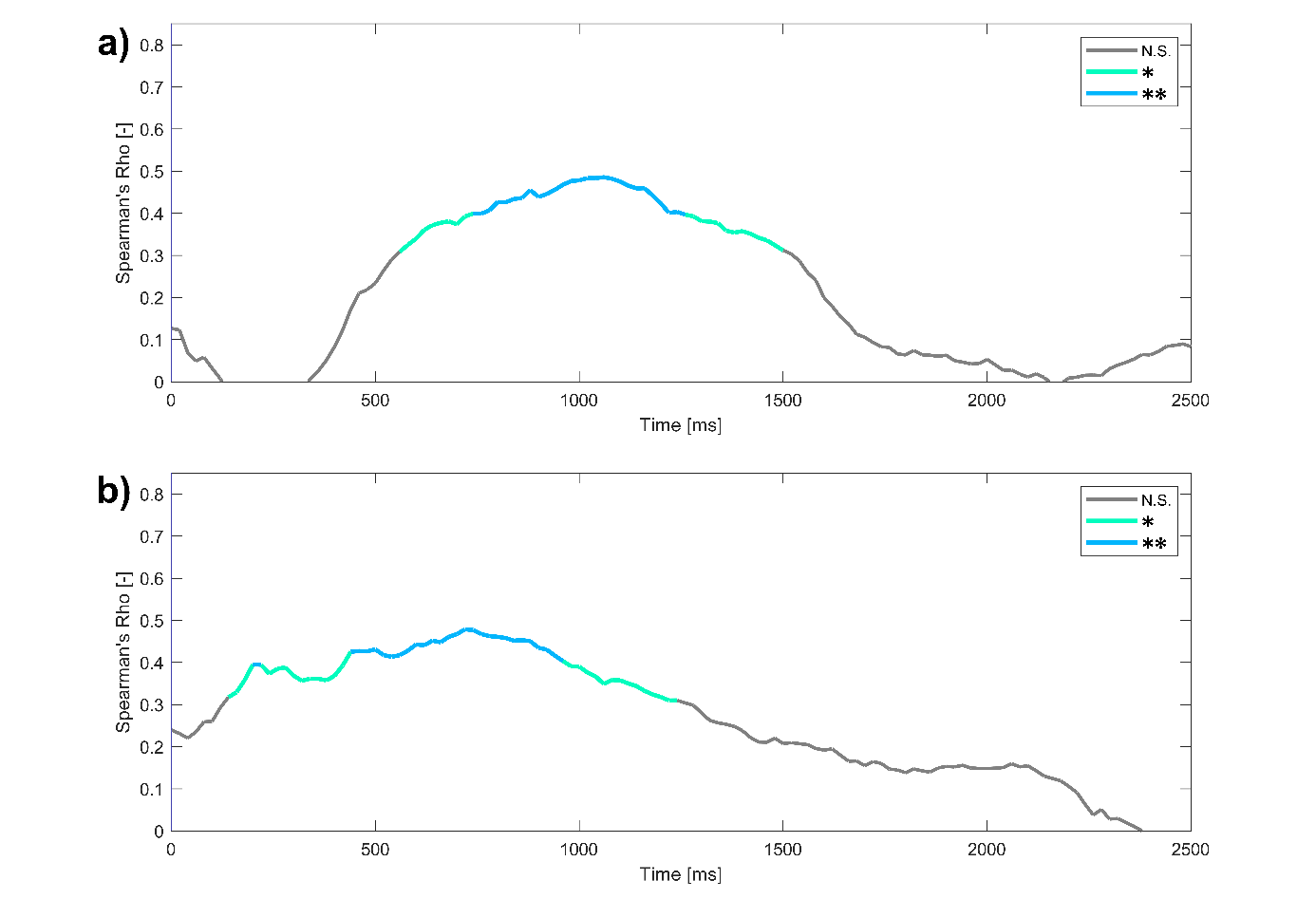


*Note:* For time-course analyses, each baseline-corrected timepoint of a specific TEPR curve in session 1 was correlated with sensitivity (*d*_L_) scores, across all participants (*N* = 42).

*: p < .05

**Supplementary Figure S5:**

Time-course analysis of individual TEPR curves regarding (a) target hit trials and (b) correct rejection trials in experiment 2.


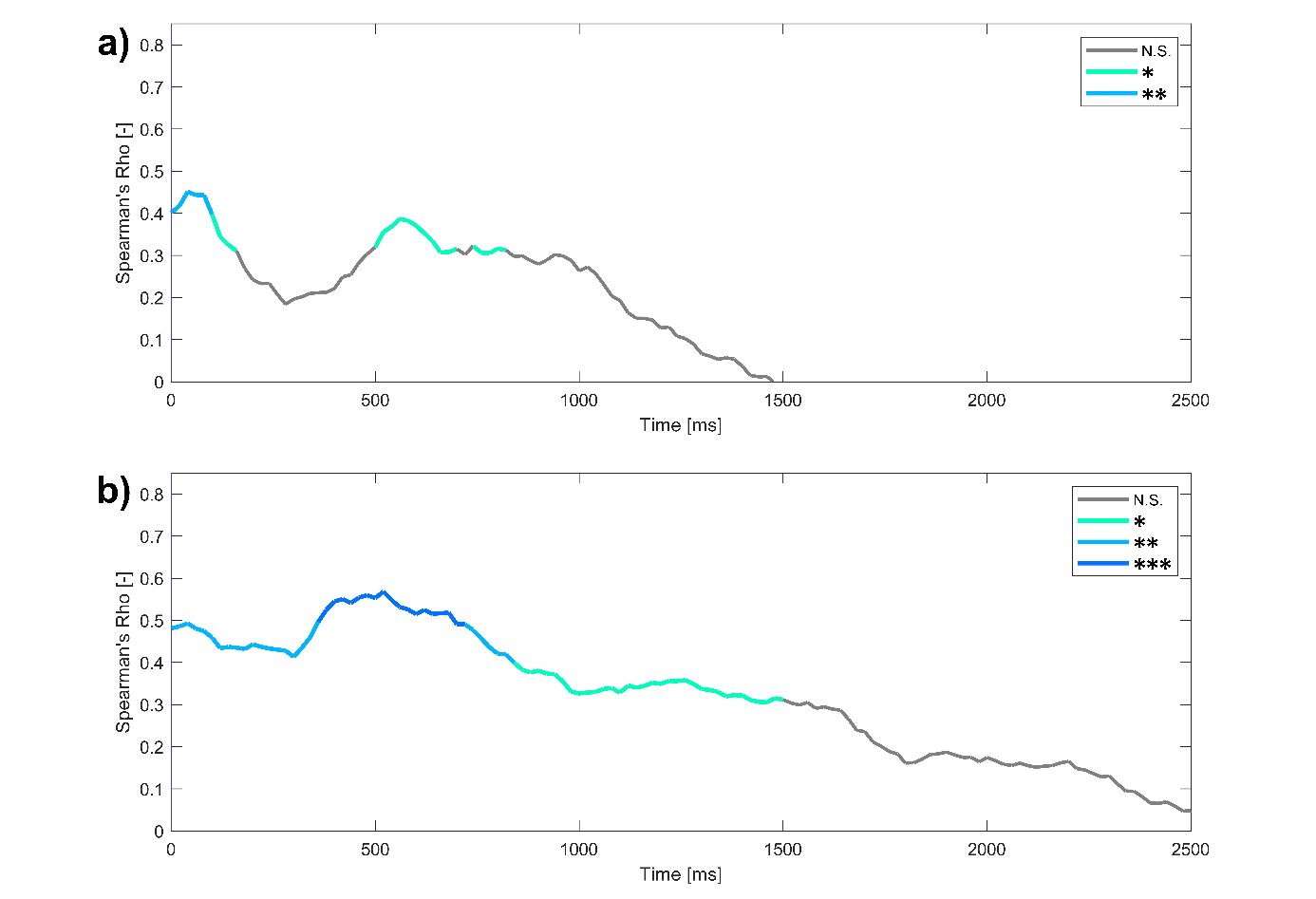


*Note:* For time-course analyses, each baseline-corrected timepoint of a specific TEPR curve in session 2 was correlated with sensitivity (*d*_L_) scores, across all participants (*N* = 42).

*: p < .05; **: p <.01; ***: p < .001

**Supplementary Figure S6:**

Time-course analysis of individual TEPR curves regarding (a) target hit trials and (b) correct rejection trials in experiment 2.


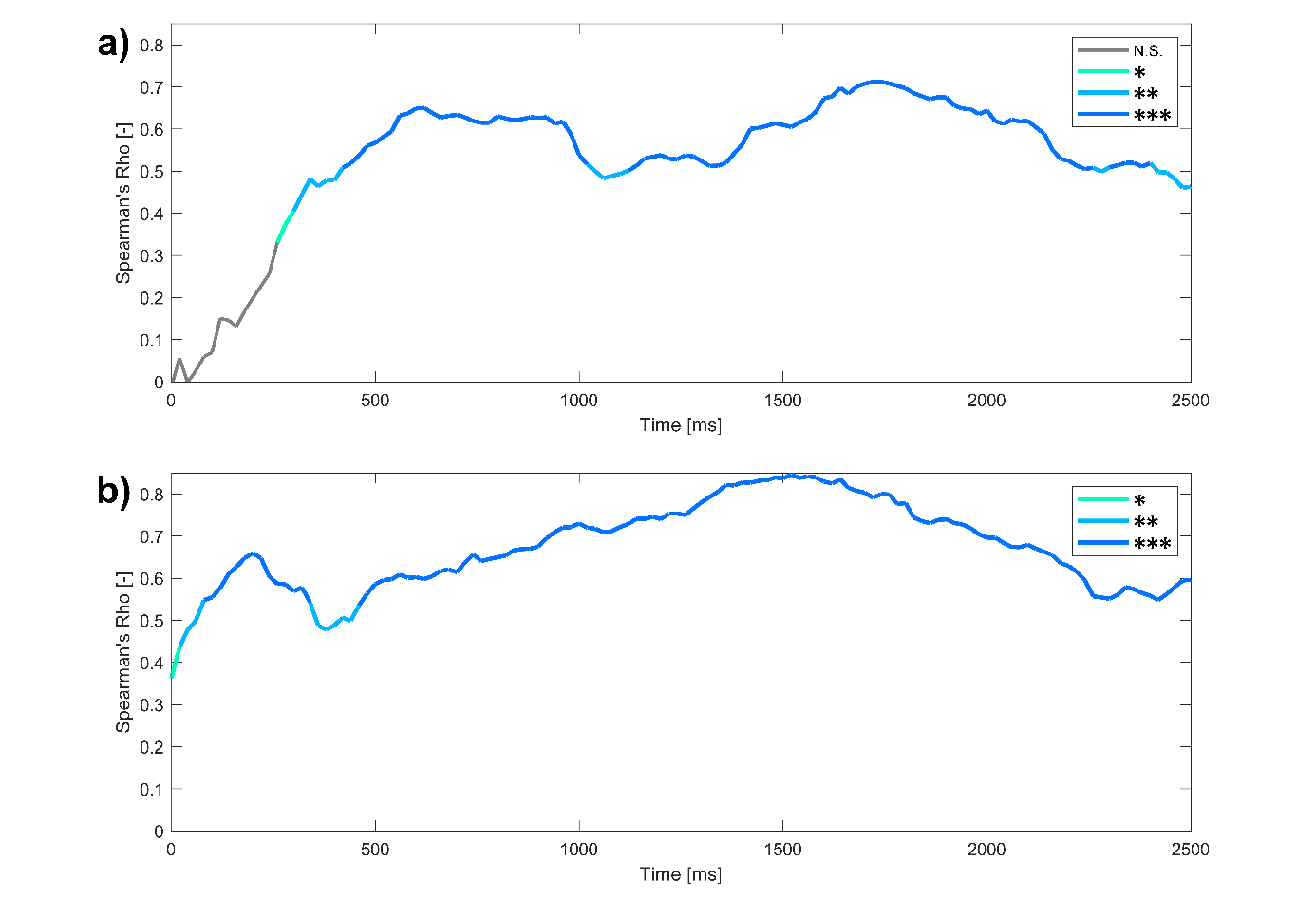


*Note:* For time-course analyses, each baseline-corrected timepoint of a specific TEPR curve in session 1 was correlated with its corresponding timepoint on session 2, across all participants (*N* = 40).

*: p < .05; **: p <.01; ***: p < .001

**Supplementary Figure S7:**

Correlations of pre-experimental and pre-trial baseline mean pupil sizes in (a) session 1 and (b) session 2, in experiment 2.





*Note:* Pupil size metrics are in millimeters. (*N* = 40)

| **Supplementary Table 1** | | | | |  |  |  |  |  |  |  |  |  |  | |  |
| --- | --- | --- | --- | --- | --- | --- | --- | --- | --- | --- | --- | --- | --- | --- | --- | --- |
| *Descriptive Statistics* | | | | | | | | | | | | | | |  |  |
|  |  |  |  |  |  |  |  |  |  |  |  |  |  |  | |  |
|  |  |  |  |  |  |  |  |  |  |  |  |  |  |  | |  |
|  |  | Session 1 | | | | | |  | Session 2 | | | | | | |  |
| Metric |  |  | Pre-trial baseline mean | Pre-trial baseline CV | Hit  Peak | CR  Peak |  |  |  |  | Pre-trial baseline mean | Pre-trial baseline CV | Hit  Peak | CR  Peak | |  |
|  |  |  |  |  |  |  |  |  |  |  |  |  |  |  | |  |
|  |  |  |  |  |  |  |  |  |  |  |  |  |  |  | |  |
| N |  | 64 | | | | | |  | 63 | | | | | | |  |
| M |  |  | 4.931 | 0.069 | 0.172 | 0.010 |  |  |  |  | 4.807 | 0.076 | 0.199 | 0.033 | |  |
| SD |  |  | 0.694 | 0.031 | 0.120 | 0.075 |  |  |  |  | 0.738 | 0.030 | 0.152 | 0.082 | |  |
| Min |  |  | 3.540 | 0.032 | -0.037 | -0.202 |  |  |  |  | 3.626 | 0.036 | -0.168 | -0.168 | |  |
| Max |  |  | 6.995 | 0.154 | 0.503 | 0.190 |  |  |  |  | 6.683 | 0.169 | 0.572 | 0.197 | |  |
|  |  |  |  |  |  |  |  |  |  |  |  |  |  |  | |  |
| *Note*: Pupil size metrics are in millimeters. CRs: Correct rejections; CV: Coefficient of Variation | | | | | | | | | | | | | | |  | |
|  |  |  |  |  |  |  |  |  |  |  |  |  |  |  |  | |

| **Supplementary Table 2** | | | | |  |  |  |  |  |  |  |  |  |  | |  |
| --- | --- | --- | --- | --- | --- | --- | --- | --- | --- | --- | --- | --- | --- | --- | --- | --- |
| *Descriptive Statistics* | | | | | | | | | | | | | | |  |  |
|  |  |  |  |  |  |  |  |  |  |  |  |  |  |  | |  |
|  |  |  |  |  |  |  |  |  |  |  |  |  |  |  | |  |
|  |  | Session 1 | | | | | |  | Session 2 | | | | | | |  |
| Metric |  | Pre-exp. baseline mean | Pre-trial baseline mean | Pre-trial baseline CV | Hit  Peak | CR Peak |  |  |  | Pre-exp. baseline mean | Pre-trial baseline mean | Pre-trial baseline CV | Hit  Peak | CR  Peak | |  |
|  |  |  |  |  |  |  |  |  |  |  |  |  |  |  | |  |
|  |  |  |  |  |  |  |  |  |  |  |  |  |  |  | |  |
| N |  | 42 | | | | | |  | 42 | | | | | | |  |
| M |  | 5.459 | 5.542 | 0.114 | 0.282 | 0.109 |  |  |  | 5.179 | 4.362 | 0.108 | 0.273 | 0.080 | |  |
| SD |  | 0.745 | 0.671 | 0.035 | 0.116 | 0.070 |  |  |  | 0.851 | 0.785 | 0.032 | 0.102 | 0.063 | |  |
| Min |  | 3.672 | 3.174 | 0.065 | -0.003 | -0.041 |  |  |  | 3.673 | 3.101 | 0.050 | 0.049 | -0.022 | |  |
| Max |  | 7.069 | 6.621 | 0.214 | 0.605 | 0.274 |  |  |  | 7.001 | 6.321 | 0.187 | 0.618 | 0.225 | |  |
|  |  |  |  |  |  |  |  |  |  |  |  |  |  |  | |  |
| *Note*: Pupil size metrics are in millimeters. CRs: Correct rejections; CV: Coefficient of Variation | | | | | | | | | | | | | | |  | |
|  |  |  |  |  |  |  |  |  |  |  |  |  |  |  |  | |

| **Supplementary Table 3** |
| --- |
| *Mediation Analyses*   \|  \|  \|  \|  \|  \|  \|  \|  \|  \|  \| \| \| --- \| --- \| --- \| --- \| --- \| --- \| --- \| --- \| --- \| --- \| --- \| \|  \|  \|  \|  \|  \|  \|  \|  \|  \|  \| \| \|  \| Mediation Model \| \| \| Direct effect \| \| \| Indirect effect \| \| \| \| Model \| IDV (of Session 1) \| DV (of Session 2) \| Mediator  (composite score) \| Effect \| S.E. \| CI_95_ \| Effect \| S.E. \| CI_95_ \| \| \|  \|  \|  \|  \|  \|  \|  \|  \|  \|  \| \| \|  \|  \|  \|  \|  \|  \|  \|  \|  \|  \| \| \| 1. \| Sensitivity (*d*_L_) \| Sensitivity (*d*_L_) \| TEPR for Hits \| **0.88** \| **0.14** \| **0.59-1.16** \| -0.01 \| 0.29 \| -0.17-0.05 \| \| \| 2. \| Sensitivity (*d*_L_) \| Sensitivity (*d*_L_) \| TEPR for CRs \| **0.84** \| **0.14** \| **0.54-0.77** \| 0.04 \| 0.03 \| -0.05-0.17 \| \| \| 3. \| TEPR for Hits \| TEPR for Hits \| Sensitivity (*d*_L_) \| **0.58** \| **0.12** \| **0.35-0.82** \| -0.01 \| 0.03 \| -0.07-0.07 \| \| \| 4. \| TEPR for CRs \| TEPR for CRs \| Sensitivity (*d*_L_) \| **0.65** \| **0.09** \| **0.46-0.84** \| 0.04 \| 0.03 \| -0.02-0.11 \| \| \|  \|  \|  \|  \|  \|  \|  \|  \|  \|  \| \| \| *Note:* IDV: Independent variable; DV: Dependent variable; S.E.: Standard error; CI: Confidence interval; CRs: Correct rejections; Bold font marks effects with a confidence interval not containing zero \| \| \| \| \| \| \| \| \| \| \| \| \|  \|  \|  \|  \|  \|  \|  \|  \|  \|  \| \| |
|  |
|  |
